# Dynamic SUMO remodeling drives a series of critical events during the meiotic divisions in *Caenorhabditis elegans*

**DOI:** 10.1371/journal.pgen.1007626

**Published:** 2018-09-04

**Authors:** Amanda C. Davis-Roca, Nikita S. Divekar, Rachel K. Ng, Sarah M. Wignall

**Affiliations:** Department of Molecular Biosciences, Northwestern University, Evanston, IL, United States of America; Memorial Sloan-Kettering Cancer Center, UNITED STATES

## Abstract

Chromosome congression and segregation in *C*. *elegans* oocytes depend on a complex of conserved proteins that forms a ring around the center of each bivalent during prometaphase; these complexes are then removed from chromosomes at anaphase onset and disassemble as anaphase proceeds. Here, we uncover mechanisms underlying the dynamic regulation of these ring complexes (RCs), revealing a strategy by which protein complexes can be progressively remodeled during cellular processes. We find that the assembly, maintenance, and stability of RCs is regulated by a balance between SUMO conjugating and deconjugating activity. During prometaphase, the SUMO protease ULP-1 is targeted to the RCs but is counteracted by SUMO E2/E3 enzymes; then in early anaphase the E2/E3 enzymes are removed, enabling ULP-1 to trigger RC disassembly and completion of the meiotic divisions. Moreover, we found that SUMO regulation is essential to properly connect the RCs to the chromosomes and then also to fully release them in anaphase. Altogether, our work demonstrates that dynamic remodeling of SUMO modifications facilitates key meiotic events and highlights how competition between conjugation and deconjugation activity can modulate SUMO homeostasis, protein complex stability, and ultimately, progressive processes such as cell division.

## Author summary

Most cells have two sets of chromosomes, one from each parent. Meiosis is a specialized form of cell division where chromosomes are duplicated once and segregated twice, in order to generate eggs (oocytes) or sperm with only one copy of every chromosome. This is necessary so that fertilization will produce an embryo that once again contains two complete copies of the genome. Using *C*. *elegans* as a model system, we have uncovered regulatory mechanisms important for the fidelity of these meiotic divisions. *C*. *elegans* oocytes use a kinetochore-independent chromosome segregation mechanism that relies on a large protein complex that localizes to the chromosomes. These protein complexes facilitate chromosome congression during metaphase and then are released from chromosomes in anaphase and progressively disassemble as the chromosomes segregate. We find that the stability and disassembly of these complexes is regulated by a protein modification called SUMO and by competition between enzymes that localize to the protein complex to either add or remove SUMO modifications. These findings provide insight into the mechanisms by which SUMO and SUMO enzymes regulate progression through cell division and illustrate a general strategy by which large protein complexes can be rapidly assembled and disassembled during dynamic cellular processes.

## Introduction

Meiosis is a specialized form of cell division where chromosomes are duplicated once and segregated twice, in order to reduce the chromosome number by half to generate haploid gametes. In contrast to mitosis, oocyte meiosis in many species occurs in the absence of centrosomes, the microtubule organizing centers that nucleate microtubules and help to define the spindle poles. The mechanisms by which chromosomes congress and ultimately segregate on these unique acentrosomal spindles are not well understood.

*C*. *elegans* oocytes utilize mechanisms for chromosome congression and segregation that are distinct from those used in mitosis. In these cells, end-on kinetochore-microtubule attachments are not apparent, and instead microtubules associate laterally with the chromosomes [1]. Additionally, segregation is kinetochore-independent, as kinetochores are normally disassembled during early anaphase, and kinetochore depletion does not affect chromosome segregation rates [2]. Although the exact mechanism driving chromosome segregation remains controversial, it is clear that both congression and segregation depend upon a large protein complex that forms a ring around the center of each bivalent in Meiosis I (MI) and around the sister chromatid interface in Meiosis II (MII). These ring complexes (RCs) are comprised of a number of conserved cell division proteins, including the Chromosome Passenger Complex/CPC (containing AIR-2/Aurora B kinase, ICP-1, CSC-1, and BIR-1), the kinesin-4 family motor KLP-19 [1], and the kinase BUB-1 [2]. During prometaphase, KLP-19 provides chromosomes with a plus-end directed force that is thought to facilitate congression to the metaphase plate [1]. Then in anaphase, separase (SEP-1) is targeted to the RCs to cleave cohesin [3], and the RCs are released and left at the center of the spindle as the chromosomes segregate [2, 3]. Depletion of some individual RC components and/or preventing the assembly of the complex as a whole results in severe chromosome segregation defects [1–11], demonstrating the importance of this complex during meiosis. Therefore, understanding how the RC assembles and is regulated will provide valuable insights into how chromosomes are accurately partitioned in oocytes.

Although the individual contribution of each RC protein to the overall functions of the complex is not fully understood, previous studies have revealed some of the principles underlying RC assembly. Initial work demonstrated that certain components are required for others to load, with the CPC required for the proper localization of all other known RC components [1, 2, 12]. Moreover, a recent study showed that the small ubiquitin-like modifier SUMO, a reversible post-translational modification, regulates RC assembly [4]. In *C*. *elegans*, there is one SUMO ortholog (SMO-1, hereafter referred to as SUMO) that can be conjugated to target proteins by the hierarchical actions of an E1 activating enzyme, an E2 conjugating enzyme (UBC-9), and SUMO-specific E3 ligases [13]. Evidence supporting a role for SUMO in RC assembly includes the demonstration that: 1) SUMO, UBC-9, and GEI-17 (a PIAS family E3 ligase) localize to the RC, 2) RC assembly is GEI-17 dependent, 3) RC components AIR-2 and KLP-19 can be SUMOylated *in vitro*, and 4) other RC components such as BUB-1 have SUMO interaction motifs (SIMs) and can interact with SUMOylated proteins [4, 14]. These findings support the view that a network of SUMO-SIM interactions between RC proteins drives the assembly of the complex. However, much still remains to be discovered about how SUMO contributes to RC organization and function.

Importantly, the mechanisms driving RC disassembly in anaphase are even less understood. Normally, AIR-2/Aurora B leaves the RCs soon after their release from chromosomes in early anaphase and relocalizes to the microtubules [6, 11]. At the same time, the released RCs appear to lose structural integrity, since they flatten by mid anaphase and then are absent by late anaphase [2, 3]. However, we recently discovered that AIR-2 relocalization to microtubules and RC disassembly are delayed in the presence of a variety of meiotic errors, demonstrating that these processes are regulated. We also found that when the RCs remained intact, anaphase spindle morphology was altered in a manner that could potentially increase the fidelity of chromosome segregation [15]. Therefore, control of RC disassembly is a central feature of anaphase progression, making it important to understand.

Here, we provide the first detailed description of RC disassembly in *C*. *elegans* oocytes and show that this process and other critical anaphase events rely on the dynamic remodeling of SUMO modifications. We found that SUMO promotes the stability of the RC and that RC disassembly is dependent on targeting the SUMO protease ULP-1 to the structures, suggesting that ULP-1 could promote RC disassembly upon removal of the E2/E3 enzymes in early anaphase. Moreover, we found that ULP-1 is active prior to anaphase and regulates aspects of RC assembly and maintenance independent of the known role for this family of proteases in SUMO maturation. Our findings therefore demonstrate that dynamic SUMO remodeling is required for key events that facilitate anaphase progression during oocyte meiosis and also demonstrate that a balance between SUMO E2/E3 enzyme and ULP-1 protease activity can regulate the SUMOylation status and thus the stability of essential protein complexes.

## Results

### RC disassembly in anaphase is a step-wise process

**Fig 1:**
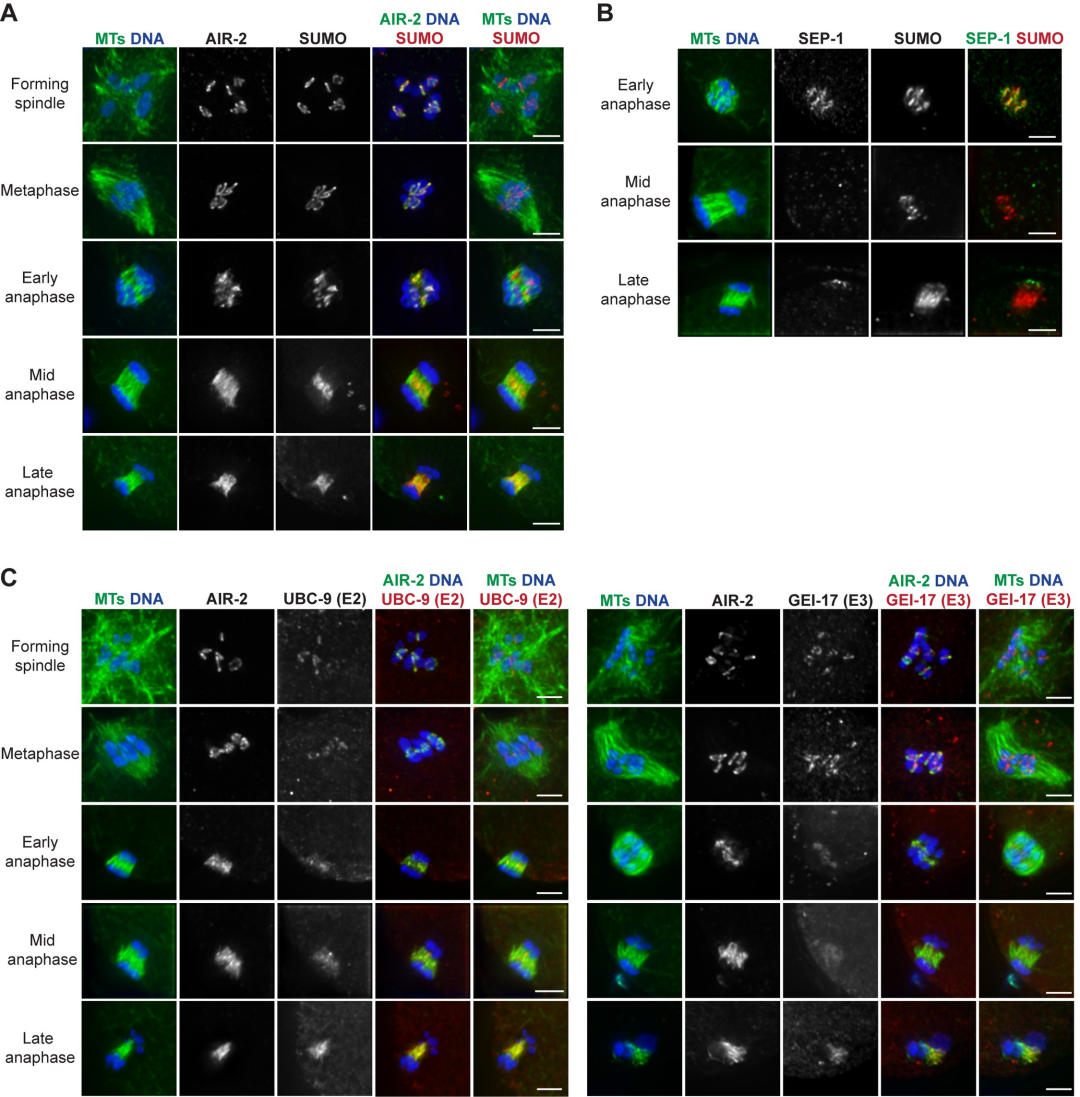
SUMO and SUMO E2/E3 enzymes leave the RCs during anaphase. (A) Localization of SUMO (red) from spindle formation through late anaphase, compared to AIR-2 (green, column 4), DNA (blue), and tubulin (green, columns 1 and 5). SUMO becomes RC associated after NEBD, but leaves the RCs by late anaphase, relocalizing to the microtubules; SUMO remains RC-associated later into anaphase than AIR-2. (B) SUMO (red), compared to SEP-1 (green, column 4), DNA (blue), and tubulin (green, column 1) throughout anaphase. SUMO remains RC-associated after SEP-1 leaves. (C) Localization of UBC-9 (SUMO E2) (red, left panel) or GEI-17 (SUMO E3) (red, right panel) from spindle formation through late anaphase, compared to AIR-2 (green, column 4), DNA (blue), and tubulin (green, columns 1 and 5). UBC-9 and GEI-17 localize to the RCs during spindle assembly and then begin to leave these structures in early anaphase. Bar = 2.5μm.

Since SUMO is RC-associated and is required for RC assembly [4], we reasoned that SUMO removal might be required for the disassembly of these complexes in anaphase. Consistent with this hypothesis, previous imaging demonstrated that SUMO leaves the RCs sometime in anaphase, relocalizing across the spindle by late anaphase [4]. However, precisely when SUMO leaves the RCs was not addressed. Therefore, we set out to carefully assess SUMO localization in relation to other RC components (Fig 1A and 1B). As shown previously, we found that SUMO is present on the RCs after nuclear envelope breakdown (NEBD) and by late anaphase had relocalized to spindle microtubules. Because a similar localization pattern is exhibited by AIR-2 [11], an RC component previously suggested to be SUMO-modified [4], we compared the behavior of these two proteins. Notably, the localization of these proteins differed in mid anaphase, with SUMO maintaining robust RC localization after AIR-2 relocalized to microtubules (Fig 1A, row 4), demonstrating that proteins other than AIR-2 are likely SUMOylated at this stage. Notably, in early anaphase spindles where a small population of AIR-2 had relocalized to microtubules, we saw that the microtubule-associated population of AIR-2 was not colocalized with SUMO (Fig 1A, row 3). These results suggest that if AIR-2 is SUMOylated when it is in the RC, this modification is either removed before AIR-2 relocalizes to microtubules or is undetectable in this small population.

We also found that SUMO persisted in the RCs longer than separase/SEP-1 (Fig 1B, row 2), demonstrating that RC components leave the complex at different times and suggesting that the disassembly of these structures is a sequential process. In addition, we confirmed that UBC-9 (SUMO E2) and GEI-17 (SUMO E3) localize to the RCs as they form (Fig 1C, rows 1–2) [4], and we observed that they remain associated with these complexes until early anaphase (Fig 1C, row 3). However, in spindles where AIR-2 had relocalized from the RCs to the microtubules (the stage at which the RCs are flattening and disassembling), the E2/E3 enzymes appeared diffuse across the spindle (Fig 1C, rows 4–5). These findings demonstrate that UBC-9 and GEI-17 removal from the RCs occurs around the time that the RCs lose structural integrity, consistent with the view that altering the SUMOylation status of the RC could play a role in disassembly.

### GEI-17-dependent SUMOylation promotes RC stability and regulates AIR-2 release from chromosomes

**Fig 2:**
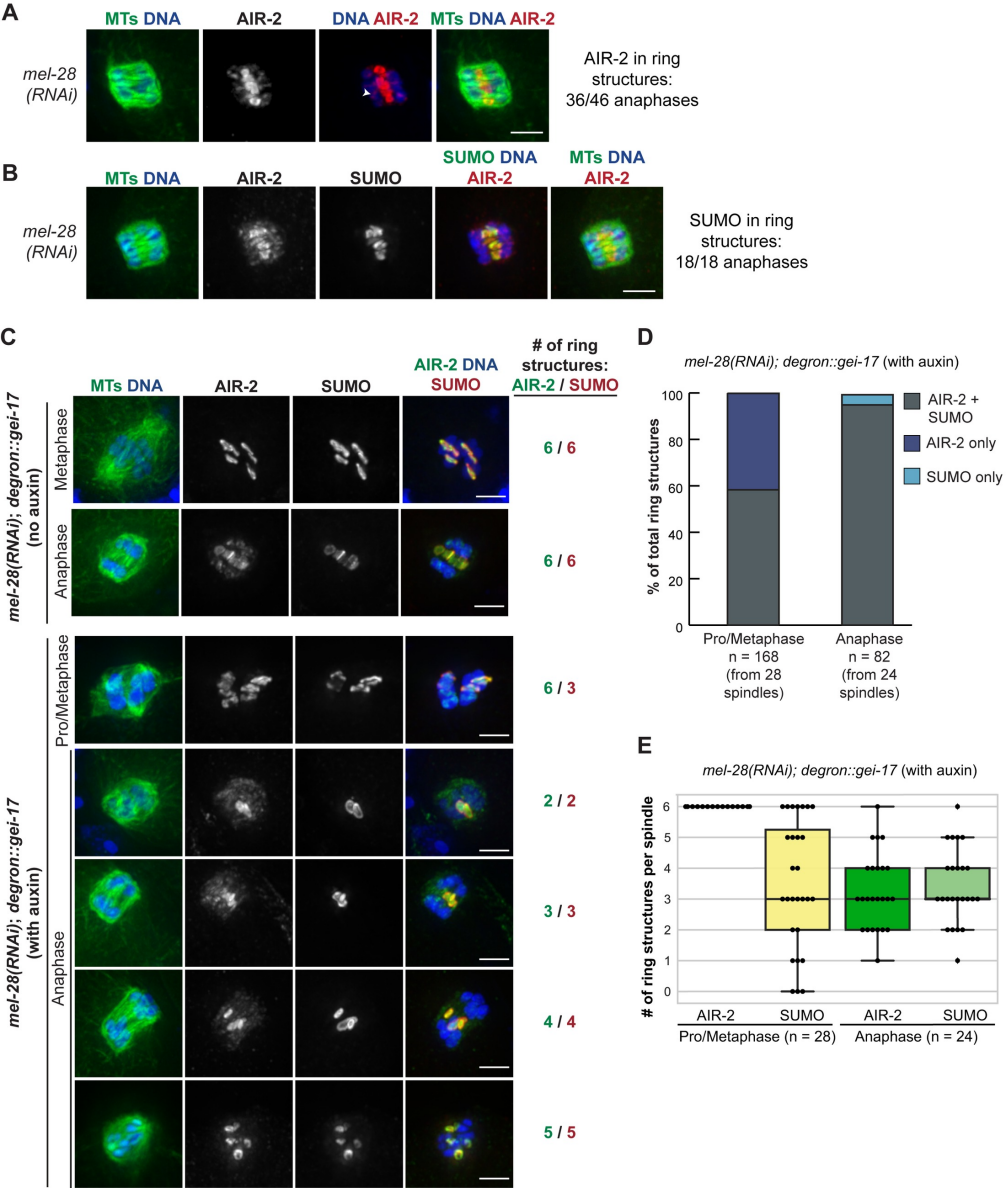
SUMO associates with stabilized anaphase RCs. (A) *mel-28(RNAi)* anaphase spindle, stained for DNA (blue), tubulin (green), and AIR-2 (red); AIR-2 remained RC-associated (36/46 anaphases observed) and RCs remained intact. Note that in many of these spindles AIR-2 had reloaded onto segregating chromosomes (arrowhead), potentially in preparation for later RC assembly in meiosis II. (B) Localization of SUMO (green, column 4), compared to AIR-2 (red), DNA (blue), and tubulin (green, columns 1 and 5) after *mel-28(RNAi)*. SUMO colocalized with AIR-2 on RCs in 18/18 spindles. (C) Spindles stained for DNA (blue), tubulin (green, column 1), AIR-2 (green, column 4), and SUMO (red). Top two rows show *mel-28(RNAi)* in the *degron*::*gei-17* strain without auxin. Six RCs marked by both AIR-2 and SUMO are present in both metaphase and anaphase. The bottom five rows show *mel-28(RNAi)* in the *degron*::*gei-17* strain after a 30 minute auxin incubation. This condition produces metaphase spindles with six RCs (shown by AIR-2 staining), of which a varying number are SUMOylated. In anaphase, the stabilized RCs always contained SUMO. Bar = 2.5μm. (D) Quantification of part C. Percent of total RCs observed in pro/metaphase or anaphase that had either both AIR-2 and SUMO present, only AIR-2 present, or only SUMO present. (E) Quantification of part C (using the same data set as part D). Number of AIR-2 stained RCs per spindle versus the number of SUMOylated RCs during pro/metaphase or anaphase. Box represents first quartile, median, and third quartile. Lines extend to data points within 1.5 interquartile range. Note that in D and E we only quantified anaphase spindles where we could distinguish at least one intact AIR-2-marked RC, excluding spindles that progressed past the *mel-28(RNAi)* arrest point due to incomplete depletion.

Given that RC disassembly appeared to be a stepwise process, we next set out to determine how early in anaphase this process was initiated. A recent study reported that following depletion of MEL-28 (a nucleoporin responsible for targeting Protein Phosphatase 1/ PP1 to meiotic chromosomes), chromosomes separate at the metaphase to anaphase transition but spindles remain in an “early anaphase” configuration, where chromosomes are unable to move very far apart [16]. We therefore asked whether the disassembly of RCs was initiated before this stage, potentially due to their physical release from chromosomes, or after. To test this, we depleted MEL-28 and then assessed the localization of AIR-2; AIR-2 is a relevant marker since it is the first known RC component to leave the RCs in anaphase, and since RC disassembly and AIR-2 relocalization are thought to occur concurrently [2, 15]. Following *mel-28(RNAi)*, we found that AIR-2 was RC-associated in the majority of anaphase spindles (36/46 spindles; 78%) (Fig 2A), demonstrating that its relocalization to the spindle is not always triggered with anaphase onset. Moreover, SUMO colocalized with AIR-2 in these structures, demonstrating that the RCs retained multiple components under these conditions (Fig 2B). These findings suggest that RC disassembly is not automatically triggered when the RCs are removed from chromosomes, and instead this process either requires MEL-28/PP1 function or relies on events after this point in early anaphase.

Notably, while the RCs usually begin to flatten out as they disassemble in mid-anaphase, we noticed that following *mel-28(RNAi)* they retained their ring-like shape (Fig 2A and 2B), suggesting that they retained structural integrity despite their removal from chromosomes. Given our hypothesis that SUMO removal from the RCs promotes RC disassembly and our finding that SUMO localizes to the *mel-28(RNAi)* stabilized RCs (Fig 2B), we reasoned that SUMO may be required for maintaining the integrity of these structures. To test this idea, we took advantage of an experimental condition we discovered that resulted in spindles with a mixture of SUMOylated and unSUMOylated RCs. We were able to achieve this using a strain in which the SUMO E3 ligase GEI-17 is linked to an auxin-inducible degron tag. Long-term depletion of GEI-17 in this strain (using a 4+ hour auxin incubation) does not affect AIR-2 chromosomal localization, but completely prevents these AIR-2-marked rings from becoming SUMOylated [4]. In contrast, we found that shorter auxin treatments resulted in spindles where some of the AIR-2-marked RCs had substantial SUMO localization while SUMO was undetectable on others; the number of SUMOylated RCs covered the whole range of zero to six per spindle (Fig 2C–2E; Supp. Fig 1, 30 minute auxin incubation shown). Therefore, acute *gei-17* depletion results in an “all or none” effect with regard to RC SUMOylation, with some RCs SUMOylated and others failing to either acquire or maintain SUMO; future experiments will be important to uncover the principles underlying this interesting switch-like behavior. However, relevant to the current study, this discovery enabled us to investigate the role of SUMO in anaphase RC stability, by combining acute GEI-17 depletion with *mel-28(RNAi)*, so that we could compare the behavior of these SUMOylated and unSUMOylated RCs during the early anaphase arrest when the RCs are normally stabilized.

Using this strategy, we found that SUMO has a role in early anaphase RC stabilization. First, while in prometaphase/metaphase there are both SUMOylated and unSUMOylated RCs, in anaphase we never observed RCs that did not contain SUMO (Fig 2C and 2D), suggesting that RCs lacking SUMO do not maintain a ring-like structure once they are released from the chromosomes. Moreover, while in prometaphase/metaphase there were always six AIR-2-marked RCs per spindle, with a variable number of these containing SUMO (average = 3.9), in anaphase the average for both AIR-2-marked structures and SUMO-marked structures was similar (average of 3.3 for AIR-2 and 3.4 for SUMO; Fig 2E), again suggesting that the RCs containing SUMO prior to anaphase onset are the only complexes that subsequently maintain their stability. We obtained similar results when we analyzed GEI-17-depleted spindles in the absence of *mel-28(RNAi)* (Supp. Fig 1), demonstrating that the stabilization of SUMO-associated anaphase RCs was not dependent upon the *mel-28(RNAi)* early anaphase arrest condition.

**Fig 3:**
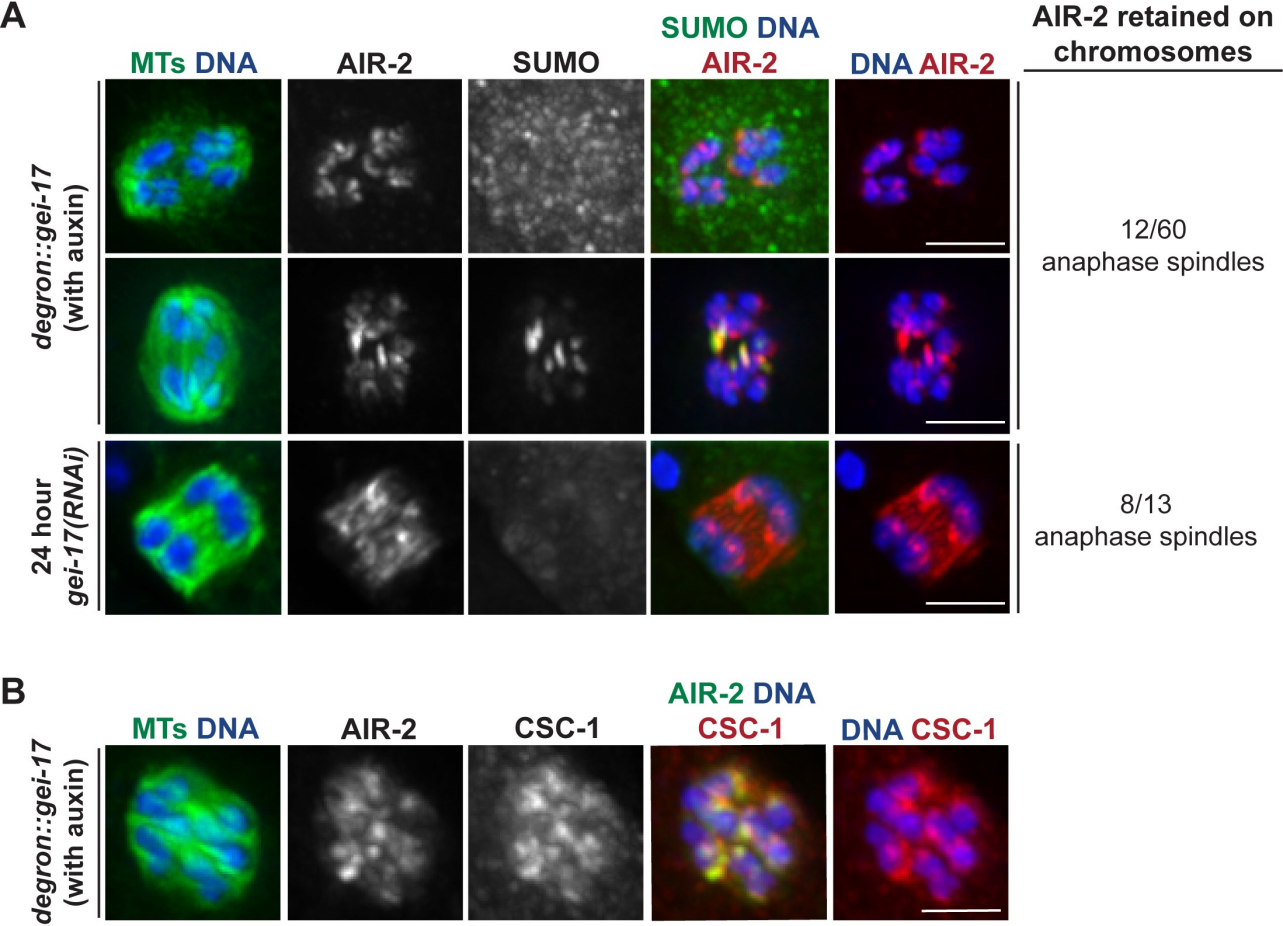
GEI-17 regulates AIR-2 release from chromosomes. (A) AIR-2 release from chromosomes is GEI-17 dependent. In both the *degron*::*gei-17* strain after a 20 minute auxin incubation and in a control strain (EU1067) after 24 hour *gei-17(RNAi)*, AIR-2 sometimes remained on inner surfaces of segregating chromosomes; this behavior did not depend on the presence of SUMO on the RC, as some of these spindles had SUMOylated RCs present and some did not. Quantification shown on right includes 20, 30, and 45-minute auxin time points. (B) CSC-1 release from chromosomes is also GEI-17 dependent. After a 20 minute auxin incubation, CSC-1 is colocalized with AIR-2 on the inner surfaces of chromosomes. Bar = 2.5μm.

During our analysis of GEI-17-depleted oocytes, we also made a surprising observation concerning AIR-2 release from chromosomes. After acute auxin-induced GEI-17 depletion, AIR-2 was retained on chromosomes in a significant number of anaphase spindles (12/60), remaining associated with the inside surfaces of separating chromosomes (a phenomenon never observed in wild-type anaphase); we also noticed this phenotype following *gei-17(RNAi)* (8/13 anaphases, 24 hour feeding RNAi used) (Fig 3A). We went on to test whether other CPC components also exhibit this retention on chromosomes after GEI-17 depletion, and we found that CSC-1 also remains chromosome-associated in anaphase, colocalizing with AIR-2 (Fig 3B). This finding is exciting because it suggests a new role for SUMOylation in CPC release from chromosomes at the metaphase to anaphase transition, and it also illustrates the dynamic and complex nature of this modification during meiotic progression.

### The SUMO protease ULP-1 is required for RC disassembly and anaphase progression

Given that SUMOylation promotes RC stability, we next set out to identify factors that could remove this modification from the RCs during anaphase. In *C*. *elegans*, there are four SUMO proteases, ULP-1, 2, 4 and 5 [14], which function to remove SUMO from target proteins [17]. Therefore, we depleted each of these proteins to assess whether any are required for RC disassembly.

First, we assessed ULP-4, since this protease was shown to regulate AIR-2 behavior in mitosis [14]. Interestingly, although ULP-4 localized faintly across the spindle and did not appear to localize to the RCs in either metaphase or anaphase, we observed some RC assembly defects upon ULP-4 depletion (S2A and S2B Fig). While AIR-2 and SUMO were targeted to the structures, they were not properly connected to the chromosomes, often appearing stretched (S2A Fig, arrow) and sometimes seeming connected to RCs on other chromosomes (S2A Fig, arrowhead). During anaphase, there were varying phenotypes; some spindles looked normal, while others had RC disassembly defects (S2A Fig, row 3) or lacked SUMO altogether (S2A Fig, row 4). These findings implicate ULP-4 deSUMOylation activity in proper RC formation and could be indicative of a role for this protease in RC disassembly. However, since it is also possible that the RC disassembly defects could be a downstream consequence of the earlier metaphase defects, these experiments do not conclusively demonstrate an anaphase role for ULP-4.

**Fig 4:**
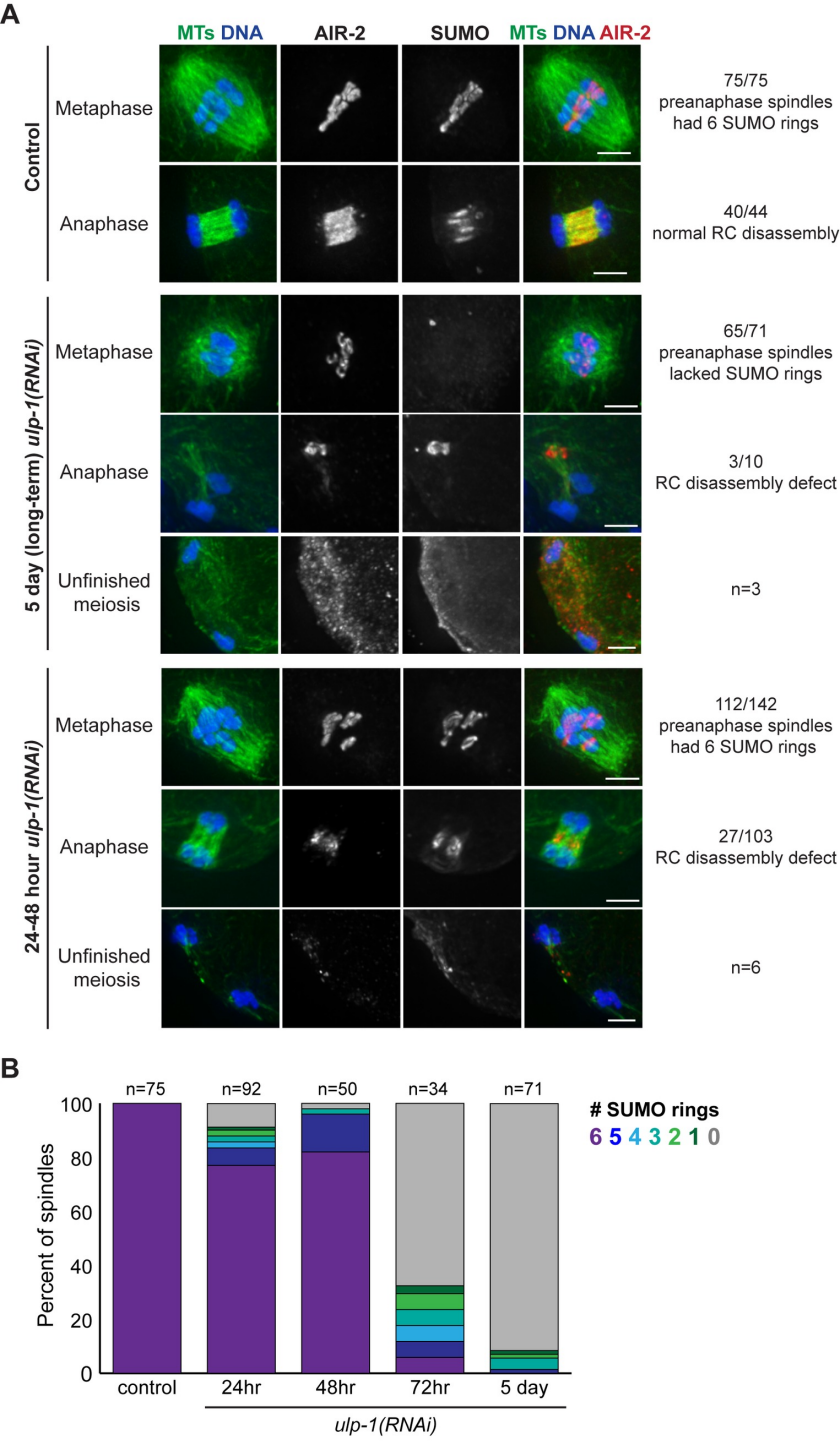
The SUMO protease ULP-1 is required for RC disassembly. (A) Spindles stained for DNA (blue), tubulin (green), SUMO (not shown in merge) and AIR-2 (red) following control, 5 day *ulp-1(RNAi)*, or 24–48 hour *ulp-1(RNAi)*. In 75/75 control pre-anaphase spindles, SUMO and AIR-2 mark 6 RCs (row 1). In 40/44 anaphase spindles, RC disassembly was normal (row 2). Following 5 day *ulp-1(RNAi)*, SUMO was not observed on the RCs in 65/71 pre-anaphase spindles (row 3). Of the 10 anaphase spindles that contained SUMO, three of the spindles had RC disassembly defects (row 4). In addition, we observed three spindles that had a failed meiosis phenotype (row 5). Following 24–48 hour *ulp-1(RNAi)*, SUMO was observed on all six RCs in 112/142 pre-anaphase spindles (row 6). Of the 103 anaphase spindles that contained SUMO, 27 of the spindles had RC disassembly defects (row 7). We also observed six spindles with a failed meiosis phenotype (bottom). Bar = 2.5μm. (B) Stacked bar graph illustrating the percent of spindles with 0, 1, 2, 3, 4, 5, or 6 SUMO rings in control spindles versus 5 days, 72, 48, and 24 hours of feeding RNAi. This analysis illustrates the sharp transition in the number of SUMOylated RCs between 48 and 72 hour depletion.

Therefore, we turned our attention to the other proteases. Depletion of ULP-2 or ULP-5 did not have obvious effects on either metaphase RC morphology, AIR-2 anaphase behavior, or RC disassembly (S2C Fig), so we did not characterize them further. Following long-term *ulp-1(RNAi*) (feeding RNAi for 5 days), we found that most spindles lacked SUMO (Fig 4A, row 3, S3A Fig, row 1), consistent with a general role for ULP-1-family proteases in processing SUMO into a conjugatable form [17, 18]. However, in some cases we observed persisting AIR-2 and SUMO structures in the center of the spindle in late anaphase (Fig 4A, row 4), suggesting that in the cases where SUMO achieved RC conjugation, RC disassembly was aberrant.

Since this result potentially implicated ULP-1 in RC disassembly, we went on to partially deplete ULP-1 using 24–48 hour feeding RNAi, rationalizing that partial ULP-1 function would promote enough SUMO processing to allow us to more specifically assess a role for ULP-1 in anaphase (Fig 4A, rows 6–8). As predicted, a majority of spindles under these depletion conditions contained six SUMOylated RCs (Fig 4B), and, consistent with our long-term depletion results, we observed instances of defective RC disassembly, with persisting structures in the center of the anaphase spindle containing AIR-2 and SUMO (Fig 4A, row 7). Other RC proteins such as BUB-1 and UBC-9 (SUMO E2) also localized to these persisting structures (S4A Fig), supporting the idea that this phenotype represents defective RC disassembly. Additionally, we also observed a small percentage of severely aberrant structures, in which chromosomes had segregated very far without extruding a polar body and SUMO and AIR-2 were faintly left behind at the center of what had been the spindle (Fig 4A, row 8); we observed this same “unfinished meiosis” phenotype occasionally in our long-term depletion experiments (Fig 4A, row 5). These results demonstrate that ULP-1 is required for RC disassembly, AIR-2 relocalization to the microtubules, and completion of the meiotic divisions. Moreover, we found that ULP-1 constructs of varying lengths can deSUMOylate both AIR-2 and KLP-19 *in vitro* (S5 Fig), two proteins previously hypothesized to be SUMOylated during RC assembly [4]. Although *in vivo* ULP-1 may have different or additional substrates, this result is consistent with the hypothesis that ULP-1 promotes RC disassembly by removing SUMO from an RC component or components.

### BUB-1 targets ULP-1 to the RC to promote disassembly

**Fig 5:**
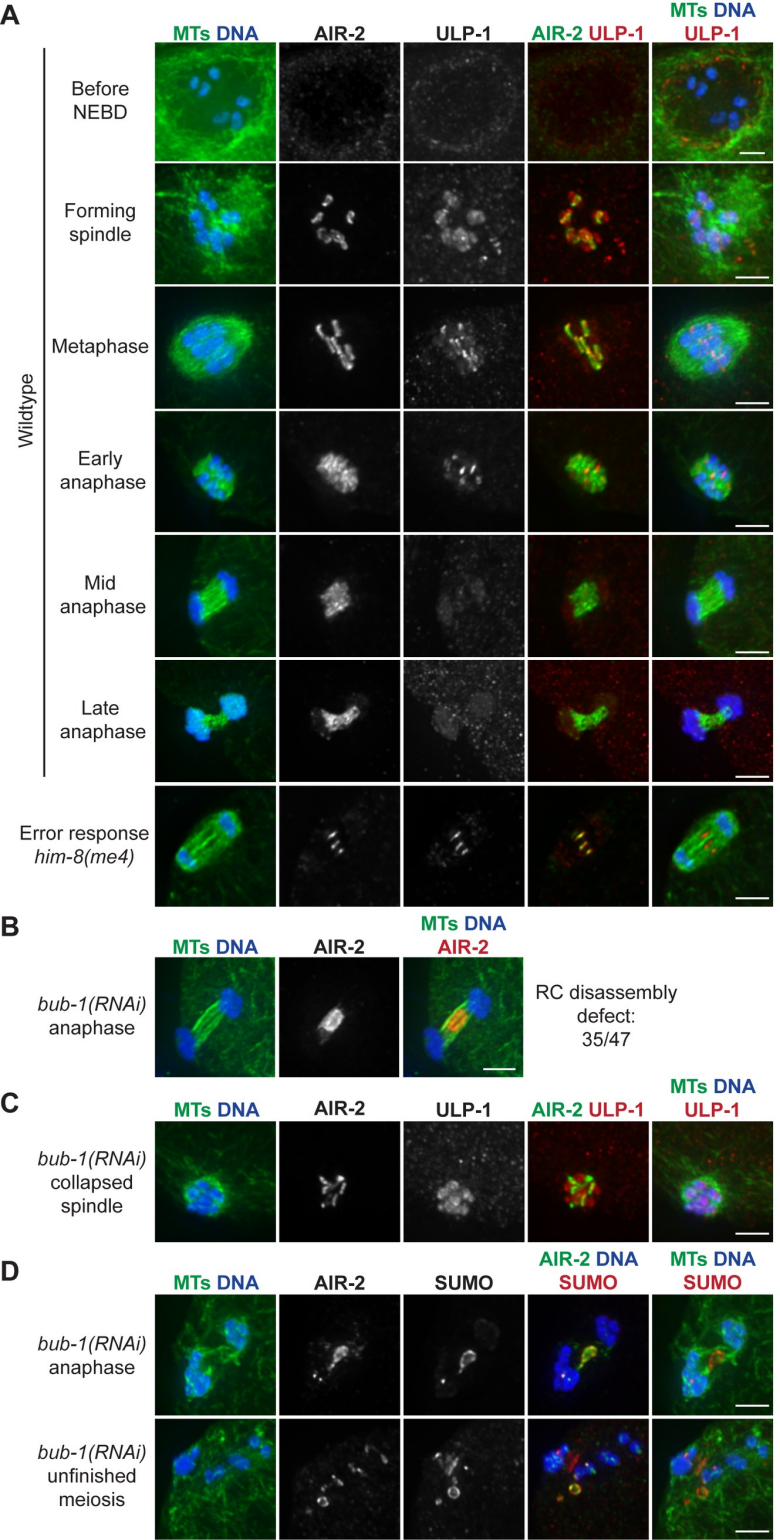
BUB-1 targets ULP-1 to the RC to promote disassembly. (A) ULP-1 (red) localization from diakinesis through late anaphase in wild-type oocytes (rows 1–6), compared to AIR-2 (green, column 4), DNA (blue), and tubulin (green, columns 1 and 5). ULP-1 begins to concentrate on the RCs during spindle formation and then leaves the RCs in mid anaphase. When RC disassembly is delayed in the *him-8(me4)* mutant, ULP-1 and AIR-2 remain associated with the RCs (row 7). (B) Anaphase spindle showing an AIR-2 (red) relocalization defect (quantification on right) after partial *bub-1(RNAi)*. Quantification of this AIR-2 relocalization defect on right includes both 48 and 72 hour *bub-1(RNAi)*. (C) Collapsed spindle following partial *bub-1(RNAi)* stained for ULP-1 (red), compared to DNA (blue), tubulin (green, columns 1 and 5), and AIR-2 (green, column 4). ULP-1 localizes to chromosomes, but is not RC-associated. (D) Partial *bub-1(RNAi)* spindles in late anaphase I (row 1) and prometaphase II (row 2) spindles, stained for SUMO (red), compared to DNA (blue), tubulin (green, columns 1 and 5), and AIR-2 (green, column 4). Defects in RC disassembly lead to persisting SUMO and AIR-2 structures. Images shown in B, C, and D include examples of 48 hour and 72 hour RNAi. Bar = 2.5μm.

Given our evidence that ULP-1 plays a role in RC disassembly, we next assessed its localization. We found that ULP-1 localizes to the nuclear envelope in oocytes during diakinesis and then becomes RC-associated after NEBD (Fig 5A, row 1–3). ULP-1 then leaves the RCs by mid anaphase, the stage at which AIR-2 has relocalized to the microtubules and the RCs have flattened and are disassembling (Fig 5A, row 5–6). ULP-1 has a similar localization pattern during MII, with additional localization to spindle poles during metaphase II (S3B Fig). These results suggest that ULP-1 is targeted to the RCs, where it could perform deSUMOylation event(s) in early anaphase to trigger disassembly. Note that we also observed a chromosomal population of ULP-1 in MI (Fig 5A, rows 2–3), but this staining was not fully removed after 5 day *ulp-1(RNAi)* (S3A Fig); this is likely due to incomplete ULP-1 depletion in our RNAi conditions, but also opens the possibility that this localization is nonspecific. We also found that ULP-1 displayed kinetochore and spindle pole localization in the one-cell stage mitotic embryo (S3C Fig), consistent with the previous demonstration that the SUMO pathway plays important roles in mitosis [14].

Next, we sought to understand how ULP-1 is targeted to the RCs. In a previous study, we assessed AIR-2 anaphase behavior in a range of depletion conditions, to characterize the response of oocytes to errors. Under most of these conditions, AIR-2 relocalization to microtubules was delayed but not prevented [15]. However, in the course of that analysis we found that depletion of RC component BUB-1 caused a severe defect in AIR-2 relocalization to microtubules, with persisting AIR-2 structures in the center of the anaphase spindle (Fig 5B), reminiscent of ULP-1 depletion (Fig 4A, row 7). This suggested that BUB-1 may be more directly involved in RC disassembly, so we investigated a possible connection between BUB-1 and ULP-1. Notably, these studies revealed that BUB-1 is required for proper ULP-1 localization; following *bub-1(RNAi)*, ULP-1 retains its broad chromosomal staining but is no longer enriched on the RCs (Fig 5C). Under these conditions, SUMO colocalizes with AIR-2 in the persisting structures at the center of late anaphase spindles during MI (Fig 5D, row 1), and these RC accumulations are also observed in the vicinity of the spindle if the oocyte is able to progress to MII (Fig 5D, row 2), similar to what we observed upon ULP-1 depletion (Fig 4A). These SUMO/AIR-2 structures also contained other RC proteins, such as CSC-1 and UBC-9 (E2) (S4B Fig), suggesting that preventing ULP-1 localization to the RCs prevents SUMO removal from these structures, consequently inhibiting RC disassembly during anaphase.

During this analysis, we noted that our *bub-1(RNAi)* conditions resulted in a more severe phenotype than our *ulp-1(RNAi)* conditions; we observed persisting AIR-2 structures in 26% of 24–48 hour *ulp-1(RNAi)* anaphase spindles (Fig 4A, row 7), compared to 75% of *bub-1(RNAi)* anaphase spindles (Fig 5B), and AIR-2 was also completely excluded from microtubules following *bub-1(RNAi)*. We speculate that the ULP-1 partial depletion conditions may allow for a small amount of active ULP-1 on the RCs. However, this difference could also indicate that BUB-1 might additionally be involved in AIR-2 regulation independent of ULP-1. Taken together, these results support the hypothesis that ULP-1 is targeted to the RCs by BUB-1, where it removes SUMO modifications in anaphase, facilitating RC disassembly.

### ULP-1 has roles on RCs independent of SUMO maturation and RC disassembly

**Fig 6:**
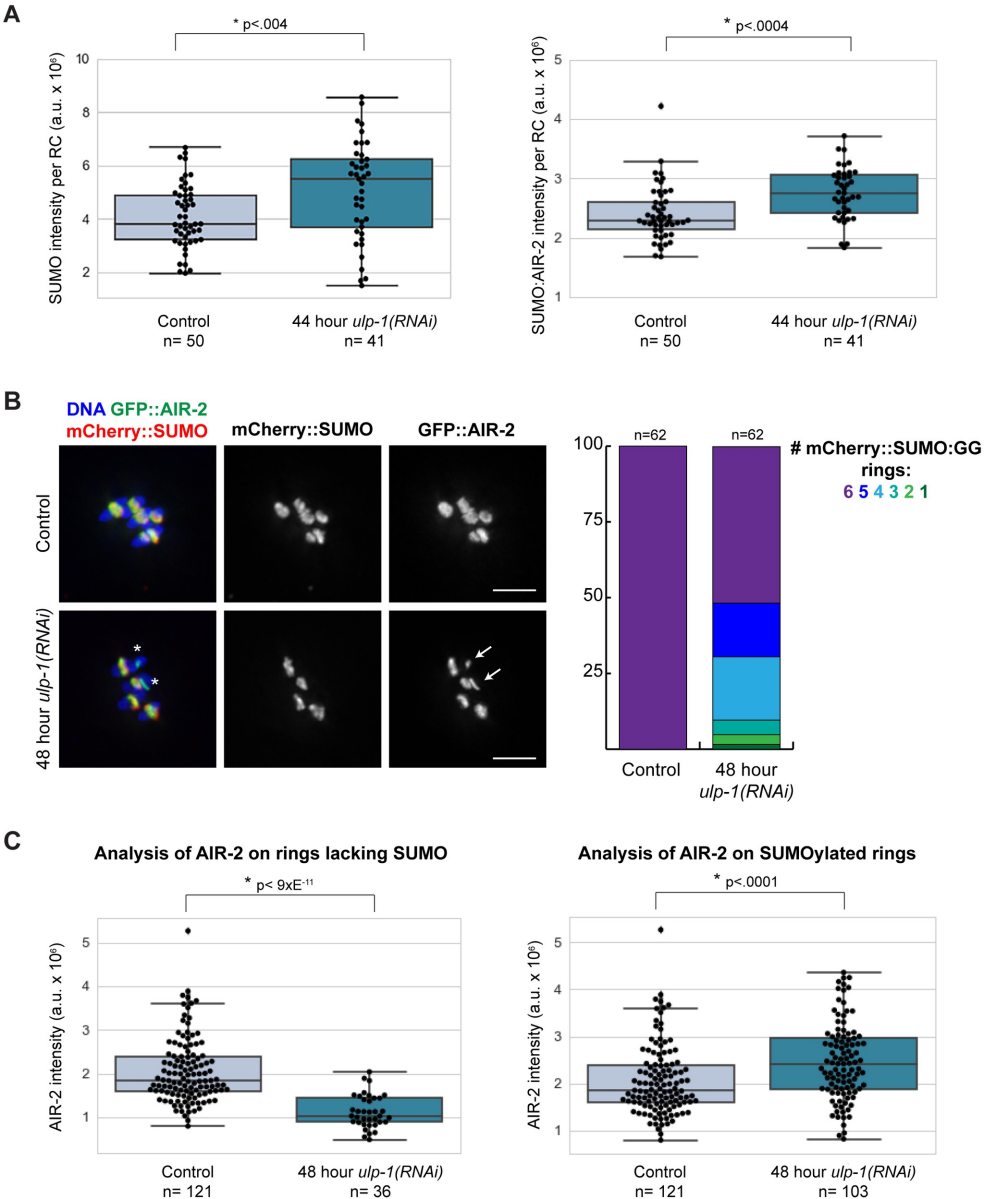
ULP-1 has a role on RCs independent of SUMO maturation and RC disassembly. (A) Box plots showing mCherry::SUMO(GG) intensity (left) or ratio of mCherry::SUMO(GG) to GFP::AIR-2 intensity (right) per RC during metaphase after control or 44 hour *ulp-1(RNAi)*. Data points in plots represent individual RCs. Box represents first quartile, median, and third quartile. Lines extend to data points within 1.5 interquartile range. Asterisks represent significant difference (two-tailed t test). Plots illustrate the significant increase in SUMO intensity per RC upon ULP-1 depletion. (B) Control versus 48 hour *ulp-1(RNAi)* metaphase chromosomes from ethanol-fixed worms expressing GFP::AIR-2 and mCherry::SUMO(GG). ULP-1 depletion results in some unSUMOylated rings (asterisks), and these rings appeared to have less AIR-2 (arrows). Percent of spindles with 1, 2, 3, 4, 5, or 6 mCherry::SUMO(GG) rings shown in stacked bar graph on the right. Bar = 2.5μm. (C) Box plots showing GFP::AIR-2 intensity per RC in control versus 48 hour *ulp-1(RNAi)* spindles. Plot on left illustrates the reduction in AIR-2 intensity on unSUMOylated RCs after ULP-1 depletion as compared to control RCs. Graph on right illustrates the increase in AIR-2 intensity on the SUMOylated RCs after ULP-1 depletion as compared to control RCs.

The finding that ULP-1 is present on RCs well before they disassemble next led us to explore the question of how ULP-1 is prevented from triggering RC disassembly prematurely. One possibility is that ULP-1 is inactive before anaphase and cannot remove SUMO from substrates at this stage. To test this hypothesis, we depleted ULP-1 and measured SUMO fluorescence intensity on the RCs prior to anaphase onset; we predicted that if ULP-1 is active then depletion of the protease would increase the amount of SUMOylation on metaphase RCs, which would be reflected by increased fluorescence. To ensure that any effects on SUMO levels were independent of a role for ULP-1 in SUMO maturation, we performed this experiment utilizing a worm strain expressing GFP::AIR-2 to mark the RCs and mCherry::SUMO(GG), a form of SUMO that can be conjugated to substrates without processing by ULP-1 [14]. Given the expression of this conjugatable form of SUMO, ULP-1 depletion should in theory not affect SUMO availability for RC assembly, enabling us to probe a role for ULP-1 independent of its SUMO processing activity. After ULP-1 depletion (via 44 hours of feeding RNAi), we found that although the GFP::AIR-2 intensity did not significantly change (S6 Fig), the mCherry::SUMO(GG) intensity per RC was increased compared to control RCs (Fig 6A, left). This result was also clear when we calculated the ratio of mCherry::SUMO(GG) to GFP::AIR-2 signal for each RC, to account for any subtle changes in the structure of particular RCs that might affect SUMO levels (Fig 6A, right). These results suggest that ULP-1 is active prior to anaphase and capable of removing SUMO from substrates at that stage. We also tested the other three ULPs using the same assay and found that SUMO intensity did not significantly change after ULP-4 and ULP-5 depletion (S7A and S7B Fig). However, ULP-2 depletion increased SUMO intensity (S7C Fig), suggesting that while this protease does not appear to be required for overall RC assembly (S2C Fig), it may more subtly regulate aspects of RC organization or function.

In the course of the above experiments, we also made an unexpected discovery that further supports a pre-anaphase role for ULP-1. When optimizing the ULP-1 partial RNAi conditions for this new strain, we noticed that longer depletions began to affect RC assembly despite the availability of conjugatable SUMO. For example, although all spindles following 48 hour RNAi contained six GFP-marked AIR-2 rings, nearly half of these spindles (30/62) had RC SUMOylation defects (Fig 6B). Reminiscent of our *gei-17* acute depletion results (Fig 2C–2E, S1 Fig), this appeared to be a switch-like effect, with mCherry::SUMO(GG) localized robustly to some RCs but undetectable on others (Fig 6B). This result further supports the idea that ULP-1 has a pre-anaphase role outside of maturing SUMO and suggests that RC-localized ULP-1 may contribute to the recruitment or maintenance of SUMOylated RC proteins.

Interestingly, our results also suggested that 48 hour ULP-1 depletion affects AIR-2 levels, as RCs lacking SUMO appeared to have less AIR-2 (Fig 6B, arrows). To quantify this effect, we measured the AIR-2 fluorescence intensity per RC after 48 hour *ulp-1(RNAi)* and found that RCs lacking SUMO showed a significant decrease in the amount of AIR-2 fluorescence intensity compared to control spindle RCs (Fig 6C, left). Additionally, the converse was also observed, with SUMOylated RCs showing an increase in the amount of AIR-2 present (Fig 6B, right). These data illustrate that despite the fact that AIR-2 initially localizes to RCs before and independent of SUMO, the progressive recruitment and maintenance of AIR-2 may be dependent on ULP-1 and/or the SUMOylation state of the RC. Taken together these results suggest that ULP-1 not only plays a role in SUMO maturation, but also has an important role on RCs prior to anaphase, both in promoting RC assembly and then also acting to maintain proper RC SUMOylation levels once the complex assembles.

### E2/E3 enzymes leave RCs before ULP-1 protease

**Fig 7:**
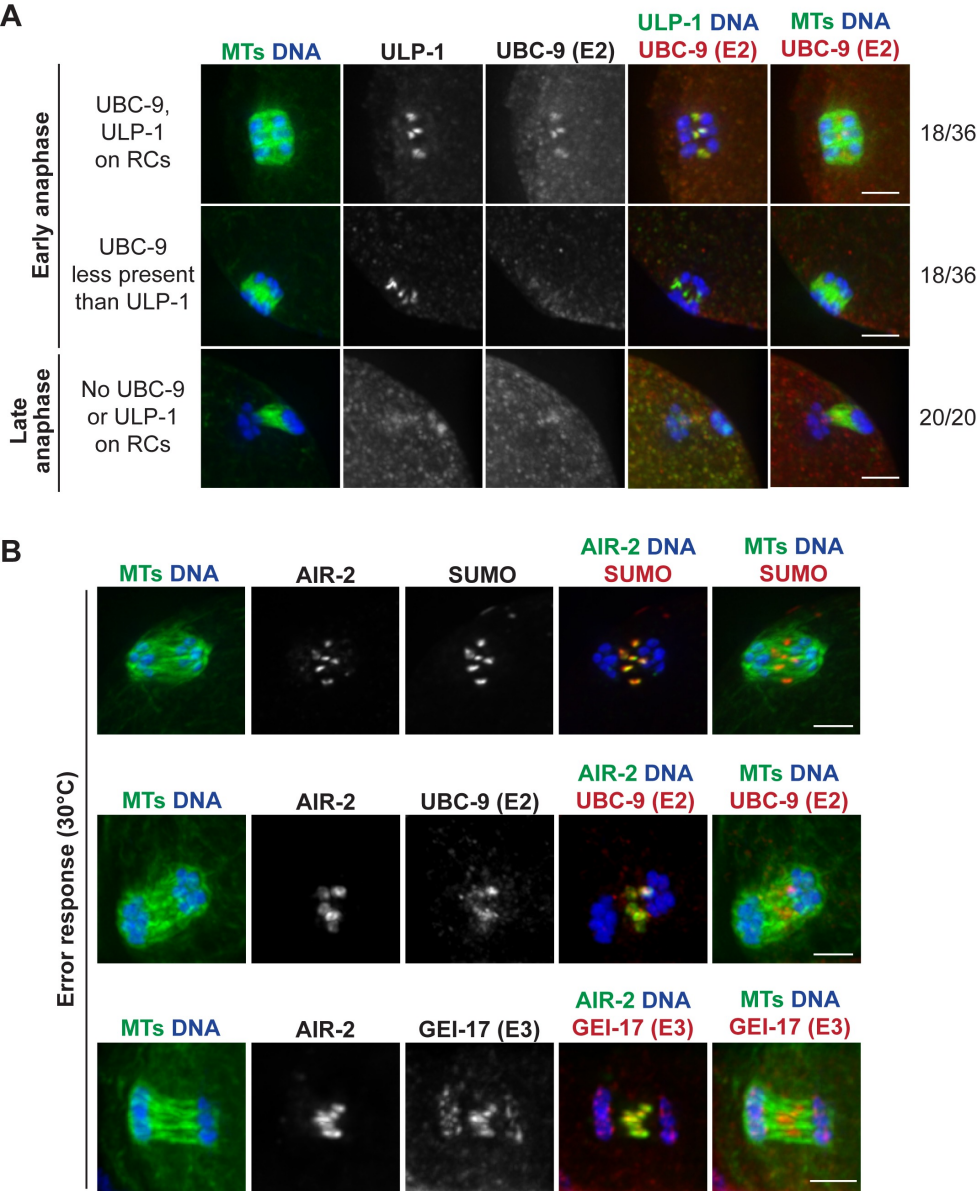
SUMO E2 UBC-9 leaves RCs before ULP-1 protease to trigger RC disassembly. (A) Comparison of SUMO E2 UBC-9 (red) to the SUMO protease ULP-1 (green in column 4), with DNA (blue) and tubulin (green, columns 1 and 5). In 50% of early anaphase spindles, UBC-9 and ULP-1 are localized to the RCs (row 1), but in the other 50%, UBC-9 is either not present or less present that ULP-1 (row 2), suggesting that conjugating enzymes leave the RCs first. As anaphase progresses, both proteins are absent from the RCs (row 3). Quantifications shown on right. (B) When RC disassembly is delayed (shown is a 30°C incubation that induces an error response), SUMO, UBC-9, and GEI-17 remain RC associated. Anaphase spindles stained with SUMO (red, top row), UBC-9 (red, second row), or GEI-17 (red, third row) and AIR-2 (green, column 4), DNA (blue), and tubulin (green, columns 1 and 5). Bar = 2.5μm.

We next hypothesized that since ULP-1 is active before anaphase and seems to compete for substrate with the E2/E3 enzymes, then removal of the E2/E3 enzymes in early anaphase could enable ULP-1 to remove SUMO modifications that trigger RC disassembly. Supporting this idea, we found that UBC-9 (E2) leaves the RCs before ULP-1 in early anaphase (GEI-17 (E3) also leaves the RCs during this time (Fig 1C), but we could not directly compare its localization to ULP-1 for technical reasons). When we co-stained early anaphase spindles with antibodies against ULP-1 and UBC-9, we found spindles distributed equally into two categories: 1) spindles where both UBC-9 and ULP-1 were present on all six RCs or 2) spindles where there was significantly more ULP-1 than UBC-9; we never observed spindles with only UBC-9 present (Fig 7A). These results support the view that prior to anaphase, RC SUMOylation is maintained by a balance between UBC-9/GEI-17 and ULP-1 activity. Then, removal of UBC-9 and GEI-17 from the RCs in early anaphase could enable ULP-1 to deSUMOylate RC components and trigger disassembly.

We further hypothesized that if UBC-9 and GEI-17 were retained on RCs past early anaphase, the RCs would maintain their ring-like structures. To test this, we assessed whether these enzymes are retained when errors are present, since we previously demonstrated that in response to various meiotic errors and short temperature stresses, the oocyte delays AIR-2 relocalization to the microtubules and RC disassembly through mid to late anaphase [15]. Under these conditions, we found that the persisting RCs were strongly marked by SUMO, UBC-9, GEI-17 (Fig 7B), and ULP-1 (Fig 5A), the latter three of which are normally absent from the RCs during this stage (Figs 1C and 5A). This suggests that UBC-9, GEI-17, and ULP-1 are actively kept on the RCs during an error response to maintain a proper balance between conjugating and deconjugating activity, thus achieving a SUMOylation state that promotes RC stability.

## Discussion

### SUMO-mediated regulation of RC assembly and disassembly

**Fig 8:**
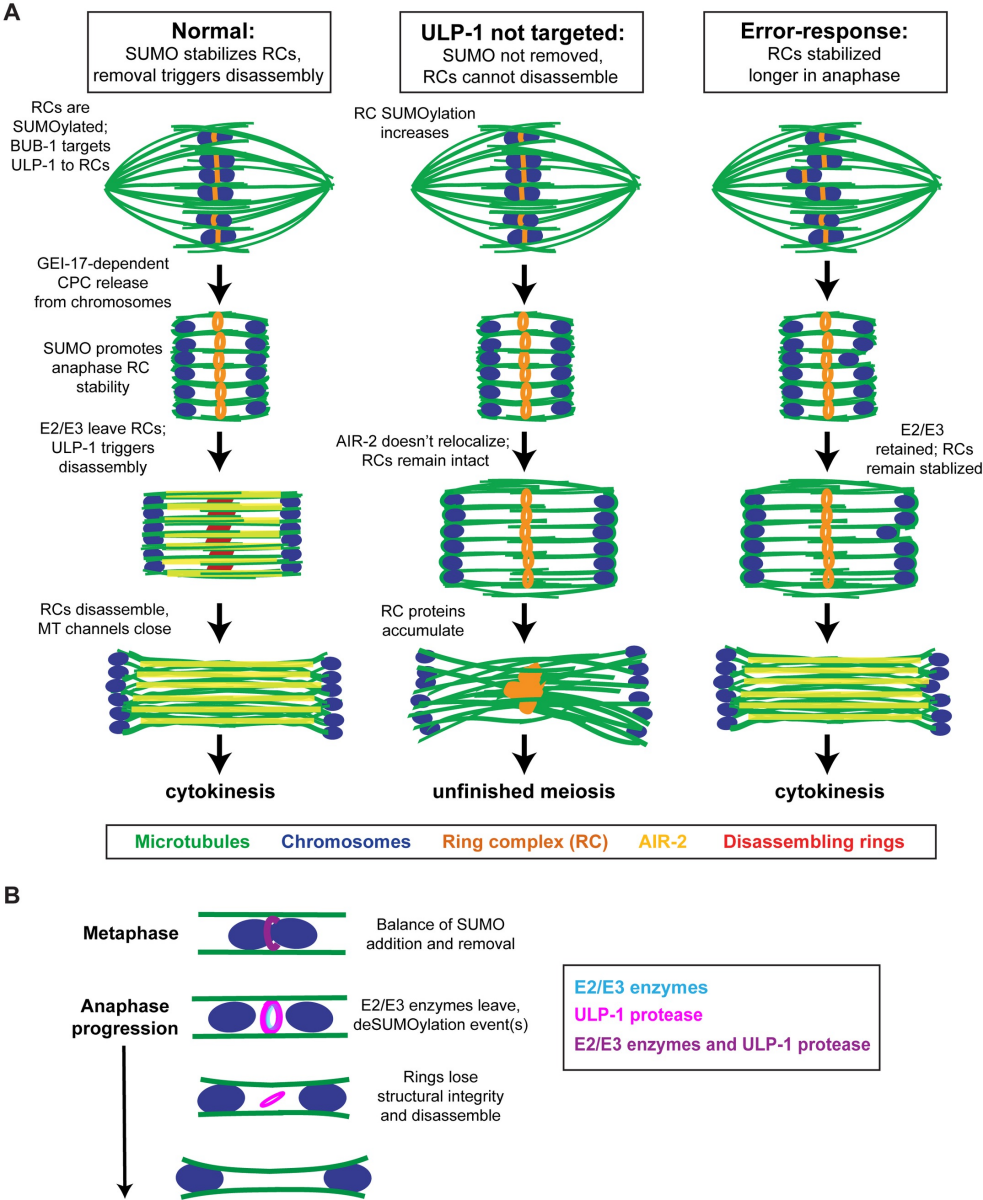
Model for SUMO-mediated anaphase regulation. (A) Model depicting DNA (blue), microtubules (green), the fully-assembled RCs with AIR-2 (orange), AIR-2 (yellow), and the disassembling RCs without AIR-2 (red). In normal oocyte meiosis (left column), the RCs are SUMOylated and the SUMO protease ULP-1 is targeted to the RCs by BUB-1. During anaphase, SUMO E2/E3 enzymes leave the RCs, allowing ULP-1 to remove SUMO modifications that trigger RC disassembly. When this happens, AIR-2 leaves the RCs and relocalizes to the microtubules and the RCs break down. Under experimental conditions in which ULP-1 cannot localize to the RC, such as ULP-1 or BUB-1 depletion (middle column), the RCs remain in a SUMOylated state that prevents RC disassembly and AIR-2 relocalization and impedes completion of the meiotic divisions. In an error-response (right column), the oocyte retains the E2/E3 enzymes to delay RC disassembly and AIR-2 relocalization. Eventually though, RC disassembly proceeds and cytokinesis occurs. (B) ULP-1 (SUMO protease, magenta), UBC-9 (SUMO E2, blue), and GEI-17 (SUMO E3, blue) are on the RCs in metaphase where they compete for substrate. During early anaphase UBC-9 (E2) and GEI-17 (E3) leave the structures and ULP-1 deSUMOylation activity promotes RC disassembly. By mid anaphase, the RCs are breaking down (not shown in cartoon) and ULP-1 is absent from the complexes.

In summary, our findings support a model in which dynamic remodeling of SUMO modifications drives a series of essential events during the meiotic divisions (Fig 8). First, SUMO is required for building the RC. SUMOylation events driven by UBC-9 and GEI-17 aid in RC assembly, enabling the targeting of other components to the structures [4]. Furthermore, we have demonstrated the importance of SUMO in promoting RC stability during anaphase. In prometaphase/metaphase, SUMO is not required for components such as those in the CPC to maintain a ring-like shape, likely since the chromosomes act as scaffolds at this stage. However, we found that SUMOylation is essential for maintaining the structural integrity of the RCs after they are released from the chromosomes, suggesting that it could act as a “glue” that provides structural support to the complex. This stabilization is important because it facilitates chromosome segregation. During early anaphase, chromosomes move towards spindle poles through microtubule channels [3, 19]. Our previous work suggested that the RCs act as physical wedges within these channels, keeping them wide to allow chromosome movements during Anaphase A and also during an error response [15]; for this function, maintaining the stability and ring-like structure of the RCs is likely important.

Our findings also demonstrate that RC disassembly in anaphase is an active process in which proteins are removed in a sequential manner from the RCs; this process is not automatically triggered when RCs are released from chromosomes and instead may rely on MEL-28 and/or PP1 function. Moreover, we have implicated deSUMOylation of RC component(s) as a major driving force. During prometaphase, ULP-1 is recruited to RCs by BUB-1 where it seems to compete with the E2/E3 enzymes for substrate. Then, at anaphase onset, removal of E2/E3 enzymes could shift this balance and allow ULP-1 to remove SUMO modifications that initiate the disassembly process. This would allow for AIR-2 relocalization to microtubules and for the RCs to lose structural integrity, flattening then breaking down, enabling the channels to close. Importantly, proper regulation and coordination of these events is essential for meiotic progression; in conditions where ULP-1 is not targeted to the RCs, RC proteins end up in persisting structures at the center of the spindle during anaphase and prevent the proper completion of the meiotic divisions.

Interestingly, our studies suggest that the disassembly process is not driven by removing SUMO entirely from the RC, since 1) the SUMO signal persists longer than ULP-1 and other RC proteins such as SEP-1 and 2) the flattening RCs still have a SUMO signal. Therefore, we propose that removal of a small population of SUMO modifications from RC components in early anaphase helps to disengage specific protein-protein interactions and allows the RC to disassemble. This could also involve deSUMOylation events that allow for Ubiquitin-mediated degradation. Since we have shown that AIR-2 and KLP-19 can be substrates of ULP-1 *in vitro*, it is possible that deSUMOylation of one of these proteins is the key event required for RC disassembly. However, since the RC is a SUMO-SIM network [4] that has at least 12 known components [1, 2, 4, 8, 9, 12, 15] and may therefore contain other SUMOylated proteins, this process is likely more complex. Future studies building on this work will therefore be important to reveal principles underlying the dynamic remodeling of SUMO-SIM networks.

### New roles for the SUMO pathway during oocyte meiosis

Our data also demonstrate that SUMO remodeling not only regulates RC assembly and disassembly but could also serve to connect the RCs to the chromosomes and regulate their release in anaphase. First, we found that depletion of the SUMO protease ULP-4 led to defective RC morphology; although ring structures still formed, they often appeared to stretch off the chromosomes and link together. This result suggests that ULP-4 is required to create a stable connection between the RCs and the chromosomes. Since ULP-4 depletion did not affect the SUMOylation level of the RCs and the protease did not appear to localize to the RC, we think that it is somewhat unlikely that ULP-4 performs this function by regulating modifications on the RCs themselves. Alternatively, we hypothesize that because ULP-4 depletion affected the level of AIR-2 on the bivalents (S7A Fig), its deSUMOylation activity instead promotes some other fundamental aspect of RC assembly. Notably, we also observed anaphase RC defects following ULP-4 depletion, raising the possibility that this protease is involved in RC disassembly alongside ULP-1. However, the metaphase RC defects that we observed made it impossible to convincingly assign an anaphase-specific role for ULP-4; future studies will therefore be important to better understand ULP-4’s precise contributions to oocyte meiosis.

Dynamic SUMO regulation also appears to be important for RC release from chromosomes. After GEI-17 depletion, we frequently observed that CPC components remained attached to chromosomes during anaphase, suggesting that release of the CPC from chromosomes is dependent on GEI-17-mediated SUMOylation. But given that AIR-2 normally loads onto chromosomes in the absence of SUMO and GEI-17, these data suggest that the SUMOylation state of the RC is remodeled during metaphase to allow for CPC release. This could be a direct modification to the CPC just before anaphase to allow it to release from histone binding, but given that SUMO does not spread to the microtubules at the same time as the CPC, in this scenario the modification would have to be quickly removed before microtubule binding in early anaphase. This idea is reminiscent of prior work demonstrating that one component of the CPC, Survivin, is ubiquitinated and deubiquitinated to promote centromere binding and release during mitosis in HeLa cells [20]. Regardless of the specific modification regulating CPC release, our results are exciting because they suggest that even after RC assembly, the SUMOylation state of the RCs continues to be modified prior to anaphase onset in order to facilitate subsequent meiotic events.

This idea that SUMO modifications are being dynamically remodeled throughout prometaphase and metaphase is also supported by our analysis of SUMO proteases. Depletion of either ULP-1 or ULP-2 increases the fluorescence intensity of SUMO on the RCs, implicating these proteases in the cleavage of SUMO from RC substrates. Moreover, ULP-1 depletion can also affect RC formation, independent of its role in maturing SUMO, demonstrating that SUMO remodeling is also likely occurring during RC assembly. These data provide further evidence that many enzymes are involved in the tight regulation of the SUMOylation states of RC proteins.

### Dynamic SUMO remodeling during cell division: An emerging view

In recent years, it has become apparent that SUMO plays an important role in the regulation of meiotic and mitotic progression, as many SUMOylated cell division proteins have been identified, SUMO localizes to the spindle in many organisms, and disruption in global SUMOylation and/or specific SUMOylated substrates generally results in severe spindle and/or chromosome segregation defects [21–29]. Given its reversible nature, SUMOylation is now appreciated as a useful post-translational modification for regulating protein localization and function during dynamic cellular processes such as cell division. Our study reinforces and expands upon this view, demonstrating a new role for SUMO in regulating anaphase progression in *C*. *elegans* oocytes by mediating the assembly and disassembly of an important protein complex. Moreover, our work reveals novel insight into the complexity of how SUMO/SIM networks can be regulated and remodeled, by targeting both SUMO E2/E3 enzymes and SUMO proteases to the protein complex to achieve fine-tuning and rapid changes in protein interactions.

In addition to further examining how competition between conjugating and deconjugating enzymes regulates the overall SUMOylation state of the RC, it will be interesting to investigate how these enzymes achieve substrate specificity. Since the RC appears to be built in discrete layers [2], one possibility is that access to substrates plays a key role in regulating substrate specificity. Additionally, phosphorylation states likely have a role in influencing protein targets, with the modification occurring on the enzyme itself and/or on the substrate RC proteins. Supporting this idea, 1) there are kinases in the RC (AIR-2, BUB-1) and 2) the phosphorylation state of many cell division proteins changes during the transition from metaphase to anaphase. Finally, many SUMO proteases have preferences for either mono-SUMOylation or various SUMO chain lengths, and this preference could act as another mode of regulation. In the future, it will be important to understand how SUMO E2/E3 enzymes and proteases act on specific substrates in order to facilitate changes in protein-protein interactions. Our study establishes the RC as an ideal model for addressing these questions, as it represents a protein complex whose progressive remodeling is regulated by a balance of SUMOylation and deSUMOylation activity. Future studies expanding upon this work will therefore not only uncover mechanisms acting to ensure the faithful segregation of chromosomes during oocyte meiosis but will also shed light on principles underlying the regulation of SUMO during dynamic cellular processes.

## Materials and methods

### Strains

Throughout this manuscript, “wild-type” refers to EU1067 worms. “Control” refers to the RNAi vector control in the corresponding worm strain. All strains used in this study are listed below.

EU1067: *unc-119(ed3) ruIs32[unc-119(+) pie-1*^*promoter*^::*GFP*::*H2B] III; ruIs57[unc-119(+) pie-1*^*promoter*^::*GFP*::*tubulin]*

FGP29: *gei-17(fgp1[GFP*::*FLAG*::*degron*::*loxP*::*gei17]) I; ieSi38[sun-1*^*promoter*^::*TIR1*::*mRuby*::*sun-1 3’UTR + Cbr-unc-119(+)] IV*

SMW6: *unc-119(ed3) ruIs32 [unc-119(+) pie-1*^*promoter*^::*GFP*::*H2B] III; ruIs57[unc-119(+) pie-1* ^*promoter*^::*GFP*::*tubulin]; him-8(me4) IV*

SMW23: *fgpls [(pFGP79) pie-1*^*promoter*^::*mCherry*::*smo-1(GG) + unc-119(+)]; ojIs50 [pie-1* ^*promoter*^::*GFP*::*air-2 + unc-119(+)]*

### RNAi

Feeding RNAi was performed as previously described [3, 15, 30, 31]. Briefly, individual RNAi clones were picked from a feeding library [32, 33] and grown overnight at 37°C in LB with 100μg/ml ampicillin. Overnight cultures were spun down, plated on NGM (nematode growth media) plates containing 100μg/ml ampicillin and 1mM IPTG, and dried overnight. Worms were synchronized by bleaching gravid adults and hatching isolated eggs overnight without food. For long-term RNAi (*ulp-1*, *ulp-2*, *ulp-4*, *ulp-5*, and *mel-28*), synchronized L1s were plated on RNAi plates and grown to adulthood at 15° for 5 days. When these RNAi conditions prevented proper germline formation or global SUMOylation, shorter-term RNAi was performed (*bub-1*, *ulp-1*, and *gei-17*). For these depletions, worms were grown on regular NGM/OP50 plates and then transferred to the RNAi plate 24–72 hours before preparing for immunofluorescence (specific timepoints shown in figures and/or figure legends). Due to the inherent variability in the partial RNAi experiments, for *gei-17*, *bub-1*, and *ulp-1 (RNAi)* we have pooled multiple independent experiments (and have performed multiple time points of feeding RNAi for *bub-1* and *ulp-1)* so that quantifications shown in the figures accurately represent of all levels of depletion and degrees of phenotypic severity we observed. Note that strains can also differ in their RNAi efficiency, with strains carrying the GFP::histone transgene (present in EU1067) being particularly RNAi-sensitive [34], so we optimized RNAi conditions for each strain independently and only pooled data obtained using the same strain. We also assessed the level of depletion for different timepoints of RNAi for GEI-17 and BUB-1 by Western blot (S8 Fig). We were unable to obtain a working antibody for ULP-1 Western blots.

### Immunofluorescence and antibodies

Immunofluorescence was performed as previously described [35]. Briefly, worms were picked into a drop of M9 buffer on poly-L-lysine slides and then cut to release oocytes. Slides were frozen in liquid nitrogen for 5–10 minutes, and then the coverslip was quickly removed. Embryos were fixed for 40–45 minutes in -20°C methanol, rehydrated in PBS, and blocked in AbDil (PBS plus 4% BSA, 0.1% Triton X-100, 0.02% Sodium azide). Primary antibodies were diluted in AbDil and incubated overnight at 4°C or at room temperature for 2 hours. Secondary antibodies were diluted in AbDil and incubated for 1 hour at room temperature. Hoechst 33342 (Invitrogen) was diluted 1:1000 in PBST (PBS + 0.1% Triton X-100) and incubated for 10–15 minutes at room temperature. Slides were washed with PBST between antibody incubations and mounted in 0.5% p-phenylenediamine in 90% glycerol, 20mM Tris, pH 8.8.

The following antibodies were used for immunofluorescence: rabbit anti-AIR-2 (1:1000; gift from Jill Schumacher), rat anti-AIR-2 (1:1000) [15], rabbit anti-SEP-1 (1:350; gift from Andy Golden), mouse anti-α-tubulin-FITC (1:500; Sigma), mouse anti-SUMO (1:500), rabbit anti-ULP-1 (1:350), rabbit anti-ULP-4 (1:300 (S2B Fig row 2,3) or 1:1500 (S2B Fig row 1)), sheep anti-UBC-9 (1:800), and rabbit anti-GEI-17 (1:600) (SUMO, ULP-1, ULP-4, UBC-9, and GEI-17 antibodies were gifts from Ronald Hay). Alexa-fluor conjugated secondary antibodies (Invitrogen) were used at 1:500. CSC-1 and BUB-1 antibodies were generated by Covance in rabbits using recombinant GST-BUB-1 (amino acids 287–661) and GST-CSC-1 (full length) proteins (purification described in the next section). Serum was then affinity purified and antibodies were used at 1:1500 (BUB-1) and 1:1000 (CSC-1).

### Protein purification

His-tagged SMO-1, UBC-9, and GEI-17 were purified using previously published methods [36] except that after Ni resin purification the proteins were loaded onto a size-exclusion column (AKTA prime plus, HiLoad 16/600 Superdex 200pg), and the protein was collected and concentrated. ULP-1 catalytic domain (“ULP-1 CD”, amino acids 470–697) and a longer fragment of ULP-1 (amino acids 284–697) were made by cloning the corresponding nucleotide sequence into a 6X his-tagged modified pET vector, transforming into BL21 DE3 cells, and growing at 20°C overnight after inducing with 0.5mM IPTG. The two proteins were purified following previously published methods for the ULP-4 catalytic domain [36]. We also attempted to generate full-length ULP-1 but had difficulties with low expression levels and severe degradation.

GST-AIR-2, GST-KLP-19 (amino acids 371–1084), GST-BUB-1 (amino acids 287–661), GST-CSC-1, and GST were made using the pGEX-6P-1 vector, and by transforming into BL21 DE3 pLysS *Escherichia coli* cells. Cultures were induced with 0.2mM IPTG and grown overnight at 20°C. After harvesting, cells were resuspended in a buffer containing protease inhibitors and 1X PBS, 10mM EGTA, 10mM EDTA, 1mM PMSF, 0.1% Tween, and 250mM NaCl, lysed by sonication, centrifuged for 40 minutes at 11,000K, and the supernatant was rotated with GST resin for 1.5–2 hours. The proteins were purified using a batch method, by rotating for 10 minutes with the appropriate buffer and spinning resin down at 2100rpm. Resin was washed 3X using a buffer containing 1X PBS, 250mM NaCl, 1mM DTT, 1mM PMSF, and 0.1% Tween. Protein was eluted using a buffer containing 50mM Tris pH 8.1, 10mM reduced glutathione, and 75mM KCl and concentrated.

### SUMOylation assays

SUMOylation reactions were performed using 2mM ATP, 5μg his-SMO-1, 200nM his-UBC-9, 12.5 or 50nM his-GEI-17, and 100ng of SAE1/SAE2 (Boston Biochem). 1μg of substrate protein (GST, GST-AIR-2, or GST-KLP-19) was used for each reaction. The reactions were performed in 50mM TrisHCl pH7.5 containing 5mM DTT, 5mM MgCl_2_, and incubated for 4 hours at 37°C. Immediately following this reaction, an aliquot was removed for a gel sample, and then the deSUMOylation assays were performed by adding 1μM his-ULP-1 CD or his-ULP-1(amino acids 284–697) directly to the SUMOylation reaction tube and incubating at 37°C for an additional hour. The following antibody concentrations were used for Western blotting: mouse anti-SUMO (1:10,000; gift from Ronald Hay), rat anti-AIR-2 (1:5000), and rabbit anti-GST (1:200; Santa Cruz). Rabbit KLP-19 antibody was generated by Covance using GST-KLP-19 stalk (amino acids 371–1084), and then affinity purified and used at 1:5000.

### SUMO intensity experiments

SMW23 worms were grown at 15°C for optimal RNAi efficiency but transferred to 25°C 16 hours prior to the experiment for optimal mCherry::SUMO(GG) expression. Worms were picked into a 7μl drop of 15°C M9 on a slide, and then the M9 was wicked away. A 10μl drop of 100% ethanol was placed on the worms, the drop was evaporated, and this was repeated twice. The slide was mounted using a solution of 50% diluted Hoechst (1:1000 in M9) and 50% mounting media (0.5% p-phenylenediamine in 90% glycerol, 20mM Tris, pH 8.8). Slides were imaged within 48 hours of preparation. Every repetition had its own control performed and imaged at the same time.

Images were deconvolved, and a sum projection was made from 11 slices encompassing an entire ring structure. One to three ring structures (from different sum projections) were analyzed per image, with the limitation being that a single ring had to be separable from other rings. A 22x20 pixel box was drawn around an area encompassing the RC, and the mCherry::SUMO(GG) and GFP::AIR-2 intensity of that area was recorded. The final SUMO and AIR-2 intensity values were then calculated by subtracting a 22x20 square of background SUMO or AIR-2 intensity from the ring SUMO or AIR-2 intensity.

### RC disassembly delay / temperature stress experiments

Worms were picked into a drop of 30°C M9, incubated for 5 minutes at 30°C, and prepared for immunofluorescence as described above.

### GEI-17 auxin-inducible degron experiments

Auxin was diluted in M9 to 1mM from a 400mM stock solution on the day of the experiment. Worms were picked into a drop of 1mM auxin (or M9 for control experiments) and incubated in a humidity chamber at 15°C for 20, 30, or 45 minutes. Worms were then cut and prepared for immunofluorescence as described above.

### Microscopy

All imaging was performed on a DeltaVision Core deconvolution microscope with a 100X objective (NA = 1.4) (Applied Precision). This microscope is housed in the Northwestern Biological Imaging Facility supported by the NU Office for Research. Images were obtained at 0.2μm z-steps and deconvolved (ratio method, 15 cycles) using SoftWoRx (Applied Precision). All images in this study were displayed as full maximum intensity projections of data stacks encompassing the entire spindle structure unless otherwise noted. Although sometimes the displayed maximum projection image does not accurately show the number of RCs/chromosomes present since structures from different z-stacks can appear to merge, for our RC quantifications we analyzed the entire z-stack, examining SUMO and AIR-2 staining separately (in grayscale) and also together (as a merge) in order to accurately count and make claims about these structures. Entire stacks for two example images are shown in S1 and S2 Movies, to illustrate this quantification method.

### Statistical methods

For SUMO and AIR-2 fluorescence intensity experiments in Fig 6 and S6 and S7 Fig, all data points for a given condition were compared with the control data points using a two-tailed t test. Data distribution was assumed to be normal, but this was not formally tested.

### Worm lysate western blots

90 adult worms were picked onto empty plates, washed, and spun down in cold M9 twice. The M9 was reduced to approximately 20μl, then 20μl of 2x SDS sample buffer was added to the worms, and the sample was boiled for 10 minutes. The 40μl sample was run in a single lane. Westerns were probed with either rabbit anti-GEI-17 (1:5000), rabbit anti-BUB-1 (1:10000), or mouse anti-tubulin (1:5000) as the loading control.

## Supporting information

S1 Fig RCs lacking SUMO do not persist in anaphase. (A) Spindles stained for DNA (blue), tubulin (green, column 1 and 5), AIR-2 (red), and SUMO (green, column 4). *Degron*::*gei-17* strain after a 30 minute auxin incubation produces metaphase spindles in which unSUMOylated RCs (marked by AIR-2) and SUMOylated RCs (marked by AIR-2 and SUMO) are both present. In anaphase, only RCs containing SUMO retain their ring-like structure. Bar = 2.5μm. (B) Quantification of part A. Percent of total RCs observed in anaphase that had both AIR-2 and SUMO present, only AIR-2 present, or only SUMO present. (C) Using the same data set as part B, another quantification of part A. Number of RCs per spindle marked with either AIR-2 or SUMO during pro/metaphase versus anaphase. Box represents first quartile, median, and third quartile. Lines extend to data points within 1.5 interquartile range.
(TIF)

S2 Fig Characterization of the SUMO proteases ULP-2, ULP-4, and ULP-5. (A) Examples of forming, metaphase, and anaphase spindles after *ulp-4(RNAi)*. Spindles stained for DNA (blue), tubulin (green, column 1), SUMO (green, column 4) and AIR-2 (red). ULP-4 depletion causes RC defects during prometaphase, with these structures appearing stretched away from the chromosomes (arrow) or merging together (arrowhead), and some anaphase defects, ranging from persisting SUMO/AIR-2 structures, to spindles lacking RC-associated SUMO. (B) Spindles stained for DNA (blue), tubulin (green), and ULP-4 (red). ULP-4 appears diffuse across the spindle, sometimes colocalized with microtubules or appearing kinetochore-like. Row 2 is a projection of 3 z-slices. (C) *ulp-2(RNAi)* and *ulp-5(RNAi)* do not have observable RC or spindle defects during metaphase or anaphase. Bar = 2.5μm.
(TIF)

S3 Fig ULP-1 antibody specificity and ULP-1 Meiosis II and Mitosis localization. (A) Spindles stained for ULP-1 (red), SUMO (green, column 4), DNA (blue), tubulin (green, columns 1 and 5) after 5 day (long-term) *ulp-1(RNAi)*. Long-term *ulp-1(RNAi)* usually prevents SUMOylation of the RCs (top), but in the rare case that the RCs are SUMOylated and formed (bottom), the ULP-1 antibody does not recognize the RCs, confirming the specificity of its localization. (B) ULP-1 localizes to the RCs and the spindle poles during metaphase II. The chromosomal localization of ULP-1 observed during meiosis I is not present. Bar = 2.5μm. (C) Mitotic spindles stained for ULP-1 (red), DNA (blue), and tubulin (green). ULP-1 localizes to metaphase chromosomes/kinetochores and spindle poles and this localization decreases in anaphase. Bar = 5μm.
(TIF)

S4 Fig Other RC components localize to the persisting SUMO and AIR-2 structures. (A) Spindles stained for UBC-9 (red, top row) or BUB-1 (red, bottom row), SUMO (green, column 4), DNA (blue), tubulin (green, columns 1 and 5) after 48 hour *ulp-1(RNAi)*. Quantification of colocalization with persisting SUMO structures shown on right. (B) Spindles stained for UBC-9 (red, top row) or CSC-1 (red, bottom row), SUMO (green, column 4), DNA (blue), tubulin (green, columns 1 and 5) after 72 hour *bub-1(RNAi)*. Quantification of colocalization with SUMO persisting structures shown on right. Bar = 2.5μm.
(TIF)

S5 Fig ULP-1 can deSUMOylate RC components AIR-2 and KLP-19 *in vitro*. (A) GST-KLP-19 and GST-AIR-2 can be SUMOylated *in vitro*, and the SUMO modifications are removed when ULP-1 catalytic domain (CD) or ULP-1 (amino acids 284–697) is added after the completed SUMOylation reaction. Western blots using anti-KLP-19 and anti-AIR-2 antibodies. ULP-1 protein sequence schematic shown on right. (B) Western blots using anti-SUMO antibody. GST-KLP-19 (top) and GST-AIR-2 (bottom) SUMOylation and deSUMOylation reactions from Part A. SUMO signal is absent after incubation with ULP-1 CD and ULP-1 (amino acids 284–697). (C) Western blot using anti-GST antibody shows that GST is not SUMOylated after SUMOylation and deSUMOylation reactions.
(TIF)

S6 Fig Analysis of AIR-2 intensity on RCs upon 44 hour *ulp-1(RNAi)*. Box plot showing GFP::AIR-2 intensity per RC during metaphase after vector control or 44 hour *ulp-1(RNAi)*; data points represent individual RCs. Box represents first quartile, median, and third quartile. Lines extend to data points within 1.5 interquartile range. n.s. = not significant (two-tailed t test). Plot illustrates no significant difference in AIR-2 intensity on RCs after 44 hour *ulp-1(RNAi)* as compared to control RCs.
(TIF)

S7 Fig AIR-2 and SUMO intensity on RCs following ULP depletions. (A-C) Box plots showing GFP::AIR-2 intensity per RC (left) or mCherry::SUMO(GG) intensity per RC (right) during metaphase after vector control or long-term (5 day) ULP depletions; data points represent individual RCs. Box represents first quartile, median, and third quartile. Lines extend to data points within 1.5 interquartile range. Asterisks represent significant difference, n.s. = not significant (two-tailed t test). (A) *ulp-4(RNAi)* results in increased AIR-2 intensity while SUMO intensity is similar to the control. (B) *ulp-5(RNAi)* also results in increased AIR-2 intensity, while SUMO did not significantly change. (C) *ulp-2(RNAi)* results in increased SUMO intensity on RCs while AIR-2 intensity is similar to control RCs.
(TIF)

S8 Fig Western blots for GEI-17 and BUB-1 depletion. (A) Western blot showing the reduction in GEI-17 after vector control, 24 hour *gei-17(RNAi)*, or 5 day *gei-17(RNAi)*. (B) Western blot showing the reduction in BUB-1 after vector control, 72 hour *bub-1(RNAi)*, or 5 day *bub-1(RNAi)*. Tubulin is shown as the loading control.
(TIF)

S1 Movie Movie steps through individual z-slices of S1A Fig, row 1 as an example of how AIR-2 and SUMO rings are counted. *Degron*::*gei-17* strain after 30 minute incubation with auxin produces metaphase spindles in which both unSUMOylated and SUMOylated RCs form (as marked by AIR-2 and SUMO); when stepping through the z-slices in this example, it is apparent that there are 6 AIR-2 rings but only 3 of these have SUMO. Spindles stained for DNA (blue), AIR-2 (green), and SUMO (red). For the quantification reported, individual channels were also examined in grayscale. Scale bar = 2.5μm.
(MOV)

S2 Movie Movie steps through individual z-slices of Fig 2C, row 7 as an example of how AIR-2 and SUMO rings in anaphase are counted. *Degron*::*gei-17* strain after 30 minute incubation with auxin and *mel-28(RNAi)* produces spindles in which only the SUMOylated rings remain stabilized during anaphase; when stepping through the z-slices in this example, it is apparent that there are 5 rings, marked by both AIR-2 and SUMO. Spindles stained for DNA (blue), AIR-2 (green), and SUMO (red). For the quantification reported individual channels were also examined in grayscale. Scale bar = 2.5μm.
(MOV)

## References

[1] Wignall SM, Villeneuve AM. Lateral microtubule bundles promote chromosome alignment during acentrosomal oocyte meiosis. Nature cell biology. 2009;11(7):839–44. Epub 2009/06/16. 10.1038/ncb1891 ; PubMed Central PMCID: PMC2760407. 19525937 PMC2760407

[2] Dumont J, Oegema K, Desai A. A kinetochore-independent mechanism drives anaphase chromosome separation during acentrosomal meiosis. Nature cell biology. 2010;12(9):894–901. Epub 2010/08/24. 10.1038/ncb2093 ; PubMed Central PMCID: PMC3052858. 20729837 PMC3052858

[3] Muscat CC, Torre-Santiago KM, Tran MV, Powers JA, Wignall SM. Kinetochore-independent chromosome segregation driven by lateral microtubule bundles. eLife. 2015;4 10.7554/eLife.06462 . 26026148 PMC4481507

[4] Pelisch F, Tammsalu T, Wang B, Jaffray EG, Gartner A, Hay RT. A SUMO-Dependent Protein Network Regulates Chromosome Congression during Oocyte Meiosis. Mol Cell. 2017;65(1):66–77. 10.1016/j.molcel.2016.11.001 ; PubMed Central PMCID: PMCPMC5222697. 27939944 PMC5222697

[5] Romano A, Guse A, Krascenicova I, Schnabel H, Schnabel R, Glotzer M. CSC-1: a subunit of the Aurora B kinase complex that binds to the survivin-like protein BIR-1 and the incenp-like protein ICP-1. J Cell Biol. 2003;161(2):229–36. 10.1083/jcb.200207117 . 12707312 PMC2172917

[6] Rogers E, Bishop JD, Waddle JA, Schumacher JM, Lin R. The aurora kinase AIR-2 functions in the release of chromosome cohesion in Caenorhabditis elegans meiosis. J Cell Biol. 2002;157(2):219–29. 10.1083/jcb.200110045 . 11940606 PMC1855215

[7] Csankovszki G, Collette K, Spahl K, Carey J, Snyder M, Petty E, et al Three distinct condensin complexes control C. elegans chromosome dynamics. Curr Biol. 2009;19(1):9–19. 10.1016/j.cub.2008.12.006 ; PubMed Central PMCID: PMC2682549. 19119011 PMC2682549

[8] Connolly AA, Sugioka K, Chuang CH, Lowry JB, Bowerman B. KLP-7 acts through the Ndc80 complex to limit pole number in C. elegans oocyte meiotic spindle assembly. J Cell Biol. 2015;210(6):917–32. 10.1083/jcb.201412010 ; PubMed Central PMCID: PMCPMC4576866. 26370499 PMC4576866

[9] Han X, Adames K, Sykes EM, Srayko M. The KLP-7 Residue S546 Is a Putative Aurora Kinase Site Required for Microtubule Regulation at the Centrosome in C. elegans. PLoS One. 2015;10(7):e0132593 10.1371/journal.pone.0132593 ; PubMed Central PMCID: PMCPMC4500558. 26168236 PMC4500558

[10] Laband K, Le Borgne R, Edwards F, Stefanutti M, Canman JC, Verbavatz JM, et al Chromosome segregation occurs by microtubule pushing in oocytes. Nat Commun. 2017;8(1):1499 10.1038/s41467-017-01539-8 ; PubMed Central PMCID: PMCPMC5684144. 29133801 PMC5684144

[11] Schumacher JM, Golden A, Donovan PJ. AIR-2: An Aurora/Ipl1-related protein kinase associated with chromosomes and midbody microtubules is required for polar body extrusion and cytokinesis in Caenorhabditis elegans embryos. J Cell Biol. 1998;143(6):1635–46. . 9852156 10.1083/jcb.143.6.1635 PMC2132979

[12] Collette KS, Petty EL, Golenberg N, Bembenek JN, Csankovszki G. Different roles for Aurora B in condensin targeting during mitosis and meiosis. J Cell Sci. 2011;124(Pt 21):3684–94. 10.1242/jcs.088336 ; PubMed Central PMCID: PMC3215577. 22025633 PMC3215577

[13] Broday L. The SUMO system in Caenorhabditis elegans development. Int J Dev Biol. 2017;61(3-4-5):159–64. 10.1387/ijdb.160388LB . 28621413

[14] Pelisch F, Sonneville R, Pourkarimi E, Agostinho A, Blow JJ, Gartner A, et al Dynamic SUMO modification regulates mitotic chromosome assembly and cell cycle progression in Caenorhabditis elegans. Nat Commun. 2014;5:5485 10.1038/ncomms6485 ; PubMed Central PMCID: PMCPMC4268692. 25475837 PMC4268692

[15] Davis-Roca AC, Muscat CC, Wignall SM. Caenorhabditis elegans oocytes detect meiotic errors in the absence of canonical end-on kinetochore attachments. J Cell Biol. 2017 10.1083/jcb.201608042 . 28356326 PMC5412562

[16] Hattersley N, Cheerambathur DK, Moyle MW, Stefanutti M, Richardson A, Lee K, et al A Nucleoporin Docks Protein Phosphatase 1 to Direct Meiotic Chromosome Segregation and Nuclear Assembly. Dev Cell. 2016;38:463–77. 10.1016/j.devcel.2016.08.006 27623381 PMC5094371

[17] Hickey CM, Wilson NR, Hochstrasser M. Function and regulation of SUMO proteases. Nat Rev Mol Cell Biol. 2012;13(12):755–66. 10.1038/nrm3478 ; PubMed Central PMCID: PMCPMC3668692. 23175280 PMC3668692

[18] Li SJ, Hochstrasser M. The Ulp1 SUMO isopeptidase: distinct domains required for viability, nuclear envelope localization, and substrate specificity. J Cell Biol. 2003;160(7):1069–81. 10.1083/jcb.200212052 ; PubMed Central PMCID: PMCPMC2172760. 12654900 PMC2172760

[19] McNally KP, Panzica MT, Kim T, Cortes DB, McNally FJ. A Novel Chromosome Segregation Mechanism During Female Meiosis. Mol Biol Cell. 2016 10.1091/mbc.E16-05-0331 . 27335123 PMC4985259

[20] Vong QP, Cao K, Li HY, Iglesias PA, Zheng Y. Chromosome alignment and segregation regulated by ubiquitination of survivin. Science. 2005;310(5753):1499–504. 10.1126/science.1120160 . 16322459

[21] Azuma Y, Arnaoutov A, Dasso M. SUMO-2/3 regulates topoisomerase II in mitosis. J Cell Biol. 2003;163(3):477–87. 10.1083/jcb.200304088 ; PubMed Central PMCID: PMCPMC2173648. 14597774 PMC2173648

[22] Ban R, Nishida T, Urano T. Mitotic kinase Aurora-B is regulated by SUMO-2/3 conjugation/deconjugation during mitosis. Genes Cells. 2011;16(6):652–69. 10.1111/j.1365-2443.2011.01521.x . 21554500

[23] Fernandez-Miranda G, Perez de Castro I, Carmena M, Aguirre-Portoles C, Ruchaud S, Fant X, et al SUMOylation modulates the function of Aurora-B kinase. J Cell Sci. 2010;123(Pt 16):2823–33. 10.1242/jcs.065565 ; PubMed Central PMCID: PMCPMC2915883. 20663916 PMC2915883

[24] Montpetit B, Hazbun TR, Fields S, Hieter P. Sumoylation of the budding yeast kinetochore protein Ndc10 is required for Ndc10 spindle localization and regulation of anaphase spindle elongation. J Cell Biol. 2006;174(5):653–63. 10.1083/jcb.200605019 ; PubMed Central PMCID: PMCPMC2064309. 16923829 PMC2064309

[25] Mukhopadhyay D, Arnaoutov A, Dasso M. The SUMO protease SENP6 is essential for inner kinetochore assembly. J Cell Biol. 2010;188(5):681–92. 10.1083/jcb.200909008 ; PubMed Central PMCID: PMCPMC2835930. 20212317 PMC2835930

[26] Yoshida MM, Ting L, Gygi SP, Azuma Y. SUMOylation of DNA topoisomerase IIalpha regulates histone H3 kinase Haspin and H3 phosphorylation in mitosis. J Cell Biol. 2016;213(6):665–78. 10.1083/jcb.201511079 ; PubMed Central PMCID: PMCPMC4915188. 27325792 PMC4915188

[27] Yuan YF, Zhai R, Liu XM, Khan HA, Zhen YH, Huo LJ. SUMO-1 plays crucial roles for spindle organization, chromosome congression, and chromosome segregation during mouse oocyte meiotic maturation. Mol Reprod Dev. 2014;81(8):712–24. 10.1002/mrd.22339 . 25123474

[28] Zhang XD, Goeres J, Zhang H, Yen TJ, Porter AC, Matunis MJ. SUMO-2/3 modification and binding regulate the association of CENP-E with kinetochores and progression through mitosis. Mol Cell. 2008;29(6):729–41. 10.1016/j.molcel.2008.01.013 ; PubMed Central PMCID: PMCPMC2366111. 18374647 PMC2366111

[29] Joseph J, Liu ST, Jablonski SA, Yen TJ, Dasso M. The RanGAP1-RanBP2 complex is essential for microtubule-kinetochore interactions in vivo. Curr Biol. 2004;14(7):611–7. 10.1016/j.cub.2004.03.031 . 15062103

[30] Wolff ID, Tran MV, Mullen TJ, Villeneuve AM, Wignall SM. Assembly of C. elegans acentrosomal spindles occurs without evident MTOCs and requires microtubule sorting by KLP-18/kinesin-12 and MESP-1. Mol Biol Cell. 2016 10.1091/mbc.E16-05-0291 . 27559133 PMC5063619

[31] Mullen TJ, Wignall SM. Interplay between microtubule bundling and sorting factors ensures acentriolar spindle stability during C. elegans oocyte meiosis. PLoS Genet. 2017;13(9):e1006986 10.1371/journal.pgen.1006986 ; PubMed Central PMCID: PMCPMC5614648. 28910277 PMC5614648

[32] Fraser AG, Kamath RS, Zipperlen P, Martinez-Campos M, Sohrmann M, Ahringer J. Functional genomic analysis of C. elegans chromosome I by systematic RNA interference. Nature. 2000;408(6810):325–30. 10.1038/35042517 . 11099033

[33] Kamath RS, Fraser AG, Dong Y, Poulin G, Durbin R, Gotta M, et al Systematic functional analysis of the Caenorhabditis elegans genome using RNAi. Nature. 2003;421(6920):231–7. Epub 2003/01/17. 10.1038/nature01278 . 12529635

[34] Hayashi M, Mlynarczyk-Evans S, Villeneuve AM. The synaptonemal complex shapes the crossover landscape through cooperative assembly, crossover promotion and crossover inhibition during Caenorhabditis elegans meiosis. Genetics. 2010;186(1):45–58. Epub 2010/07/02. 10.1534/genetics.110.115501 ; PubMed Central PMCID: PMCPMC2940310. 20592266 PMC2940310

[35] Oegema K, Desai A, Rybina S, Kirkham M, Hyman AA. Functional analysis of kinetochore assembly in Caenorhabditis elegans. J Cell Biol. 2001;153(6):1209–26. . 11402065 10.1083/jcb.153.6.1209 PMC2192036

[36] Pelisch F, Hay RT. Tools to Study SUMO Conjugation in Caenorhabditis elegans. Methods Mol Biol. 2016;1475:233–56. 10.1007/978-1-4939-6358-4_17 . 27631810

