# Supplementary figures and images for: Dynamic SUMO remodeling drives a series of critical events during the meiotic divisions in Caenorhabditis elegans
Source: PLoS Genet. 2018 Sep 4;14(9):e1007626. doi: 10.1371/journal.pgen.1007626 (PMC6138424; doi:10.1371/journal.pgen.1007626)

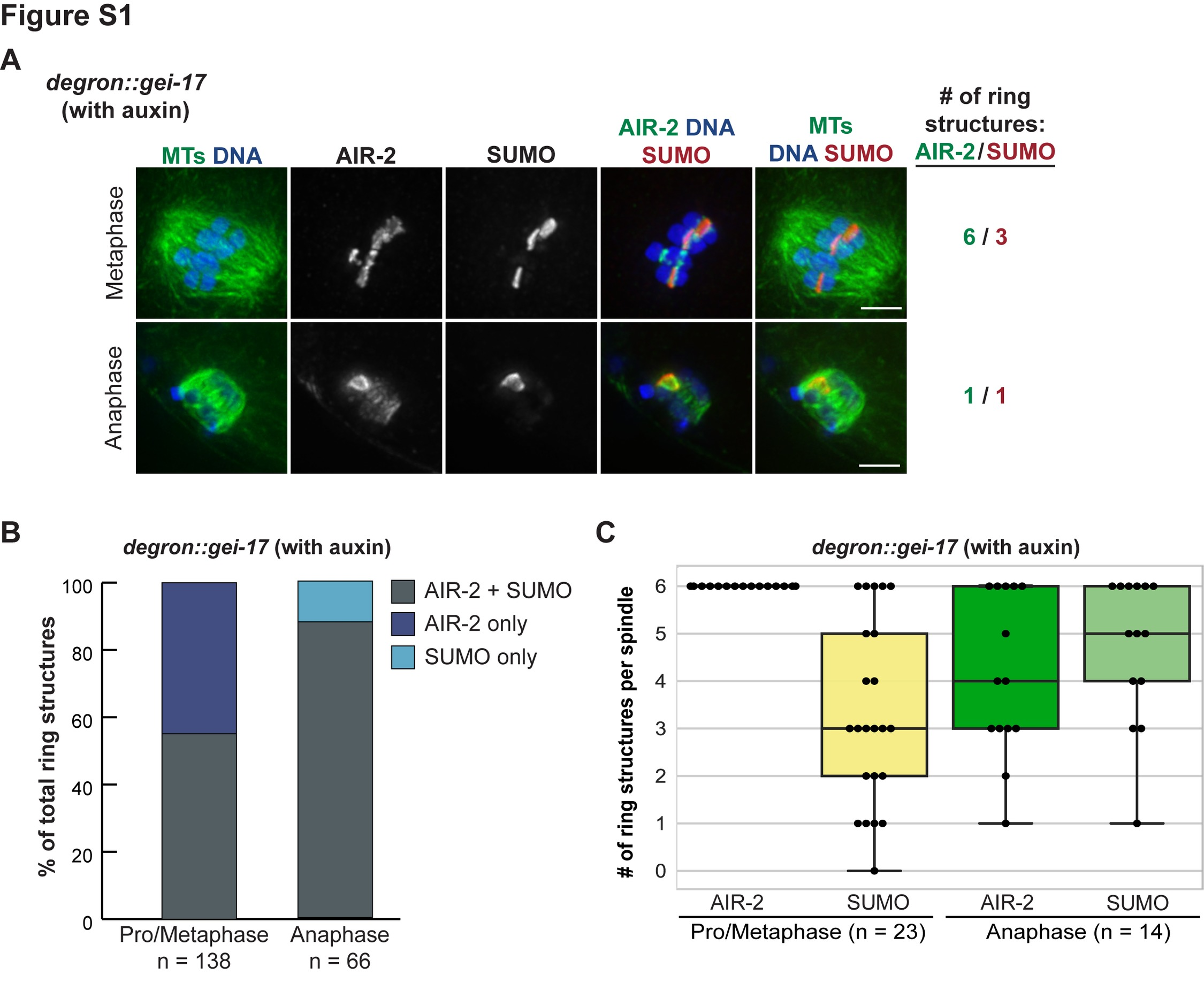

Supplement: S1 Fig — (A) Spindles stained for DNA (blue), tubulin (green, column 1 and 5), AIR-2 (red), and SUMO (green, column 4). Degron::gei-17 strain after a 30 minute auxin incubation produces metaphase spindles in which unSUMOylated RCs (marked by AIR-2) and SUMOylated RCs (marked by AIR-2 and SUMO) are both present. In anaphase, only RCs containing SUMO retain their ring-like structure. Bar = 2.5μm. (B) Quantification of part A. Percent of total RCs observed in anaphase that had both AIR-2 and SUMO present, only AIR-2 present, or only SUMO present. (C) Using the same data set as part B, another quantification of part A. Number of RCs per spindle marked with either AIR-2 or SUMO during pro/metaphase versus anaphase. Box represents first quartile, median, and third quartile. Lines extend to data points within 1.5 interquartile range. (TIF) [file pgen.1007626.s001.tif]

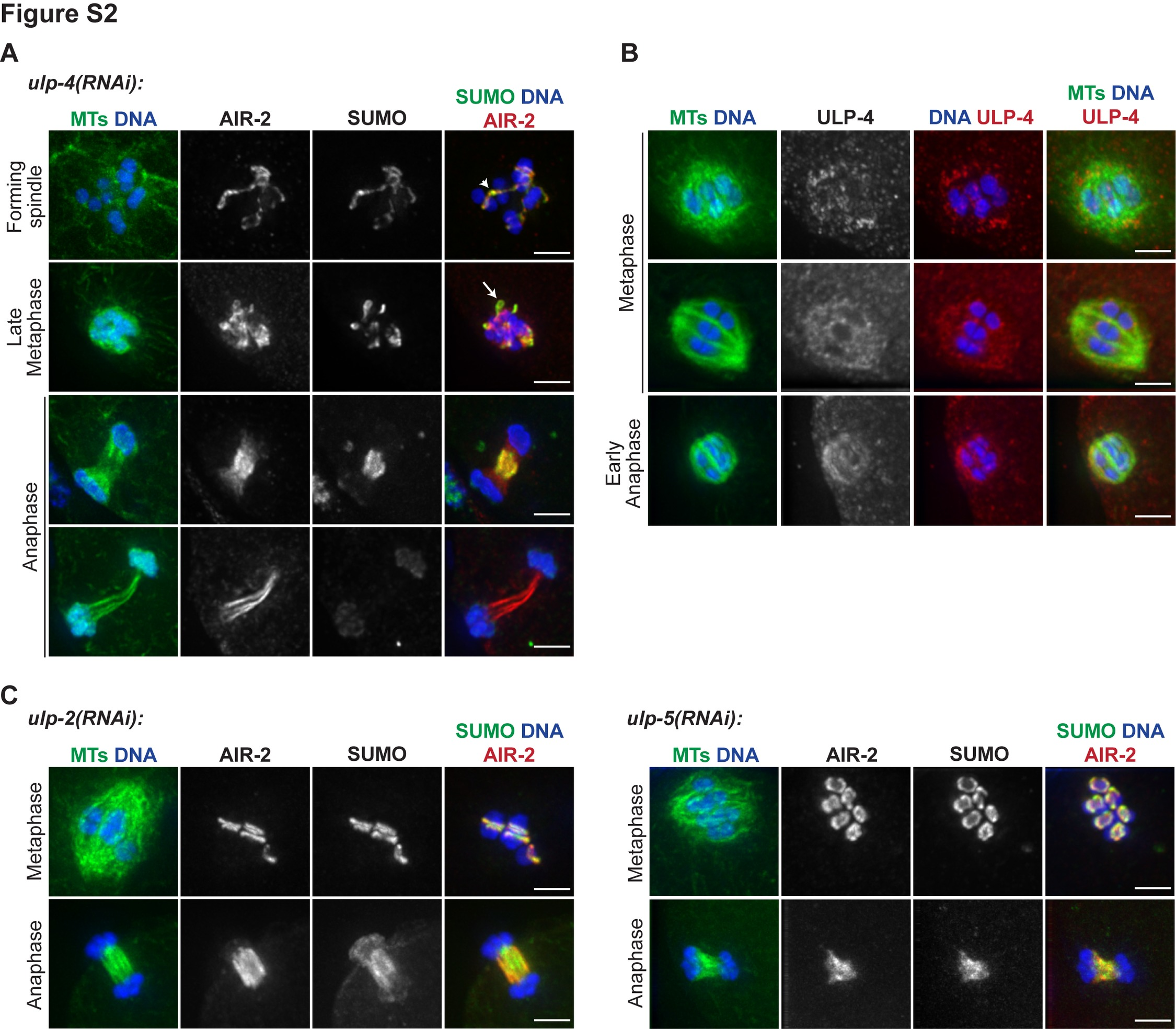

Supplement: S2 Fig — (A) Examples of forming, metaphase, and anaphase spindles after ulp-4(RNAi). Spindles stained for DNA (blue), tubulin (green, column 1), SUMO (green, column 4) and AIR-2 (red). ULP-4 depletion causes RC defects during prometaphase, with these structures appearing stretched away from the chromosomes (arrow) or merging together (arrowhead), and some anaphase defects, ranging from persisting SUMO/AIR-2 structures, to spindles lacking RC-associated SUMO. (B) Spindles stained for DNA (blue), tubulin (green), and ULP-4 (red). ULP-4 appears diffuse across the spindle, sometimes colocalized with microtubules or appearing kinetochore-like. Row 2 is a projection of 3 z-slices. (C) ulp-2(RNAi) and ulp-5(RNAi) do not have observable RC or spindle defects during metaphase or anaphase. Bar = 2.5μm. (TIF) [file pgen.1007626.s002.tif]

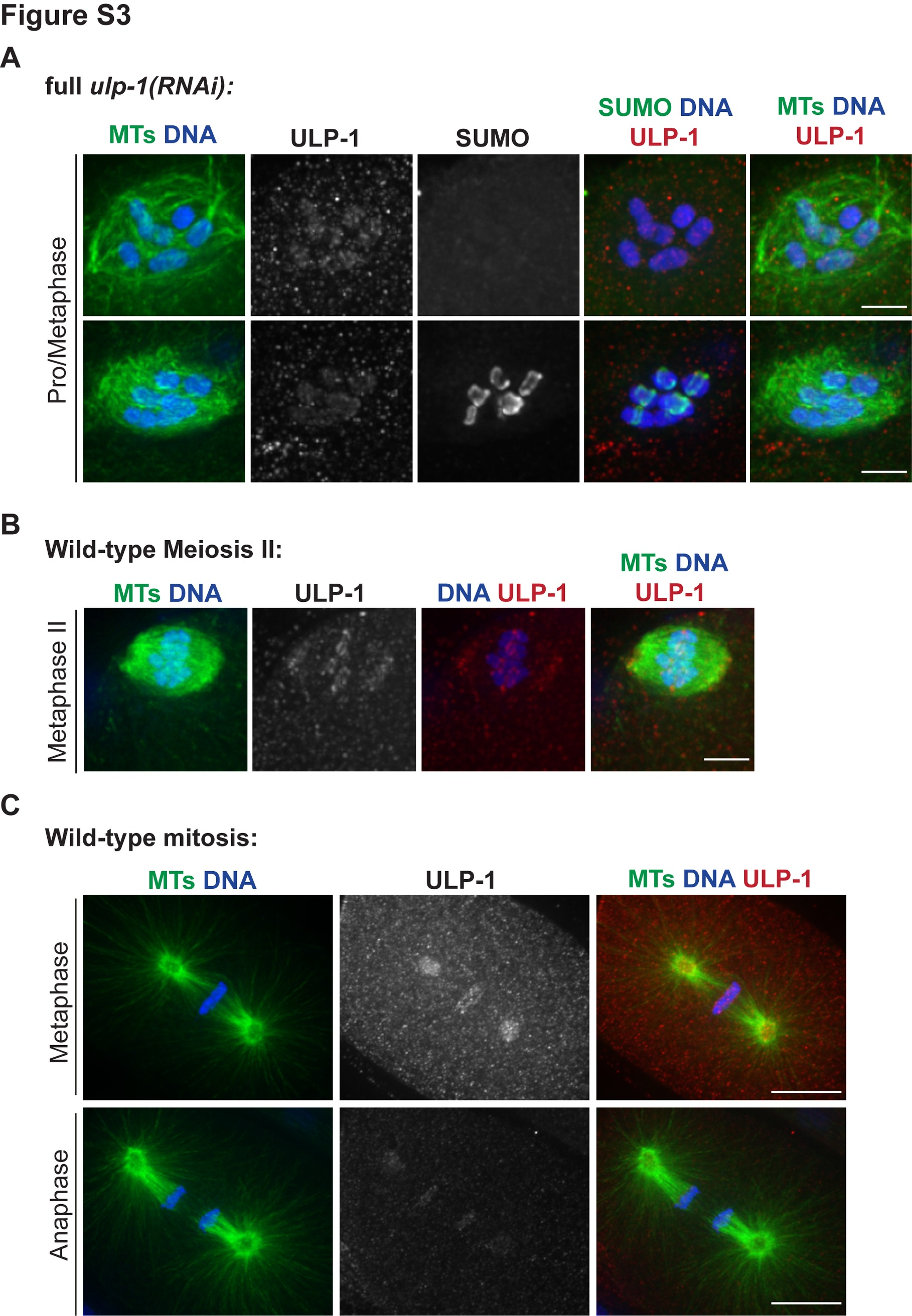

Supplement: S3 Fig — (A) Spindles stained for ULP-1 (red), SUMO (green, column 4), DNA (blue), tubulin (green, columns 1 and 5) after 5 day (long-term) ulp-1(RNAi). Long-term ulp-1(RNAi) usually prevents SUMOylation of the RCs (top), but in the rare case that the RCs are SUMOylated and formed (bottom), the ULP-1 antibody does not recognize the RCs, confirming the specificity of its localization. (B) ULP-1 localizes to the RCs and the spindle poles during metaphase II. The chromosomal localization of ULP-1 observed during meiosis I is not present. Bar = 2.5μm. (C) Mitotic spindles stained for ULP-1 (red), DNA (blue), and tubulin (green). ULP-1 localizes to metaphase chromosomes/kinetochores and spindle poles and this localization decreases in anaphase. Bar = 5μm. (TIF) [file pgen.1007626.s003.tif]

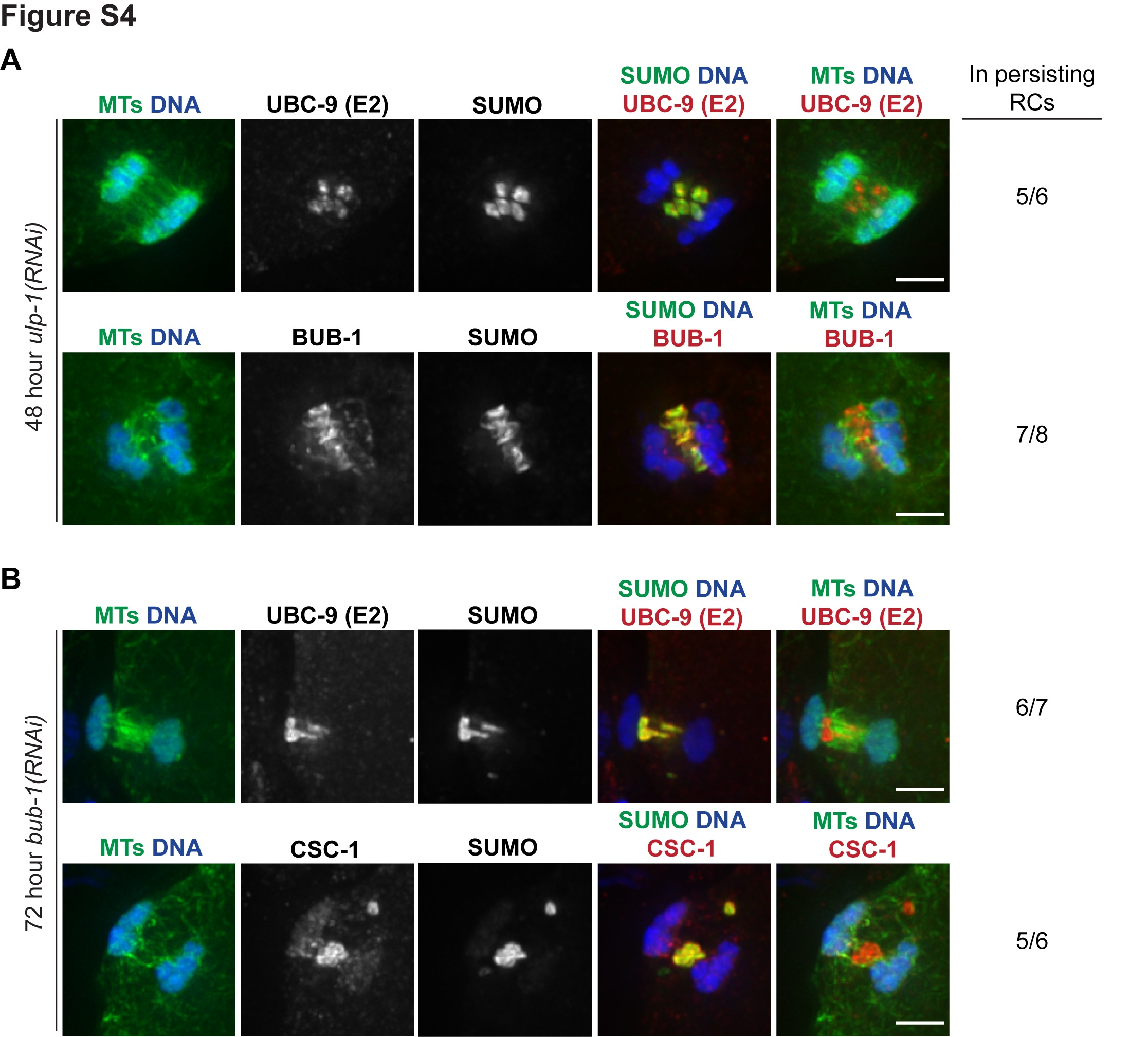

Supplement: S4 Fig — (A) Spindles stained for UBC-9 (red, top row) or BUB-1 (red, bottom row), SUMO (green, column 4), DNA (blue), tubulin (green, columns 1 and 5) after 48 hour ulp-1(RNAi). Quantification of colocalization with persisting SUMO structures shown on right. (B) Spindles stained for UBC-9 (red, top row) or CSC-1 (red, bottom row), SUMO (green, column 4), DNA (blue), tubulin (green, columns 1 and 5) after 72 hour bub-1(RNAi). Quantification of colocalization with SUMO persisting structures shown on right. Bar = 2.5μm. (TIF) [file pgen.1007626.s004.tif]

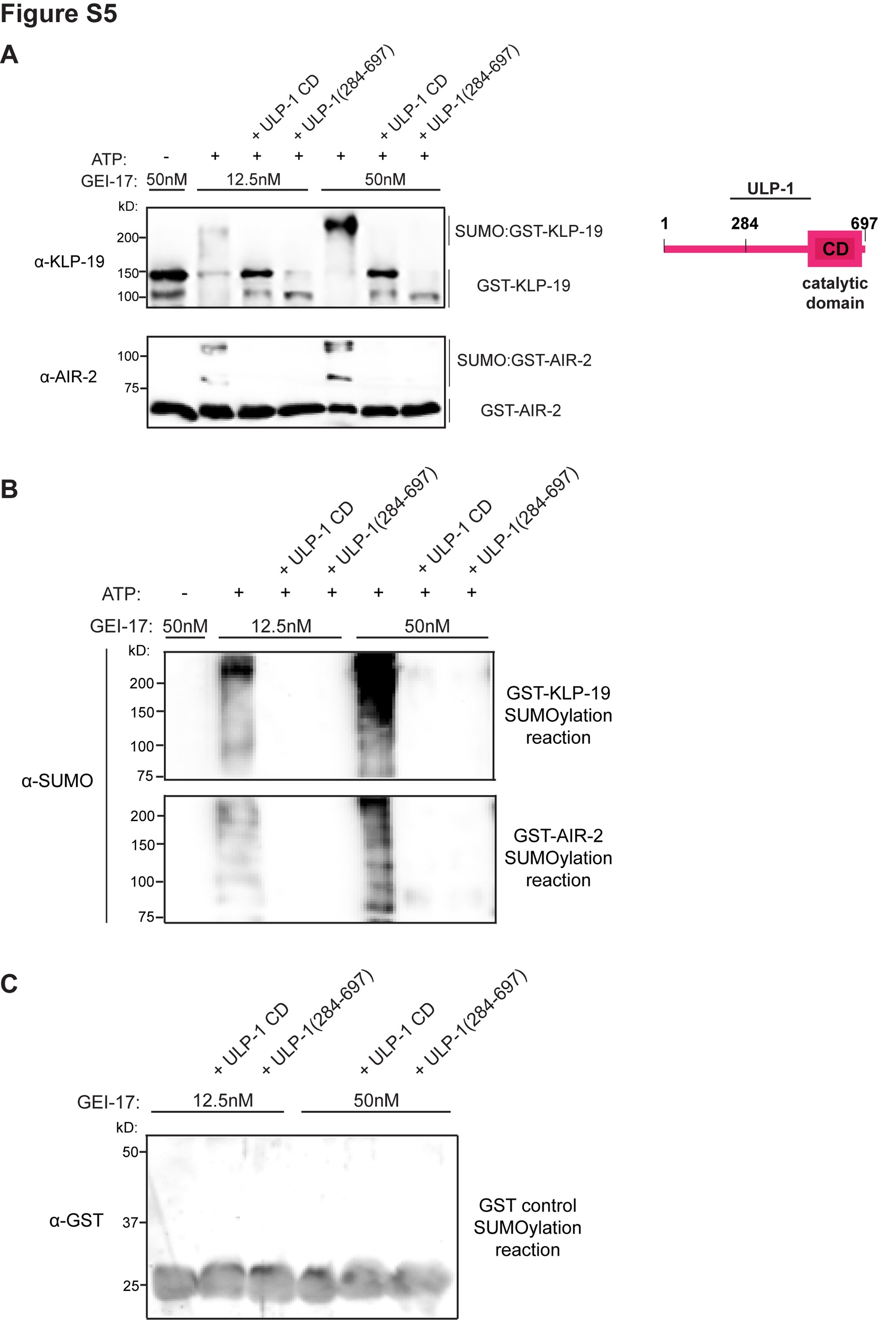

Supplement: S5 Fig — (A) GST-KLP-19 and GST-AIR-2 can be SUMOylated in vitro, and the SUMO modifications are removed when ULP-1 catalytic domain (CD) or ULP-1 (amino acids 284–697) is added after the completed SUMOylation reaction. Western blots using anti-KLP-19 and anti-AIR-2 antibodies. ULP-1 protein sequence schematic shown on right. (B) Western blots using anti-SUMO antibody. GST-KLP-19 (top) and GST-AIR-2 (bottom) SUMOylation and deSUMOylation reactions from Part A. SUMO signal is absent after incubation with ULP-1 CD and ULP-1 (amino acids 284–697). (C) Western blot using anti-GST antibody shows that GST is not SUMOylated after SUMOylation and deSUMOylation reactions. (TIF) [file pgen.1007626.s005.tif]

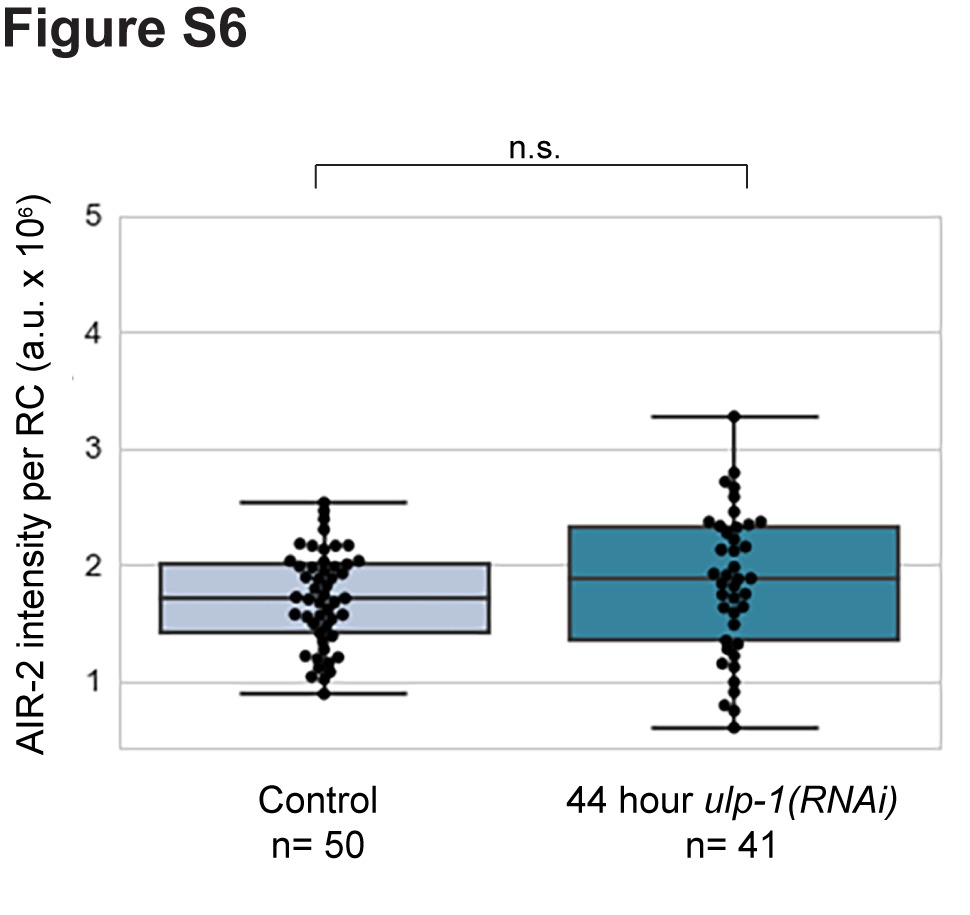

Supplement: S6 Fig — Box plot showing GFP::AIR-2 intensity per RC during metaphase after vector control or 44 hour ulp-1(RNAi); data points represent individual RCs. Box represents first quartile, median, and third quartile. Lines extend to data points within 1.5 interquartile range. n.s. = not significant (two-tailed t test). Plot illustrates no significant difference in AIR-2 intensity on RCs after 44 hour ulp-1(RNAi) as compared to control RCs. (TIF) [file pgen.1007626.s006.tif]

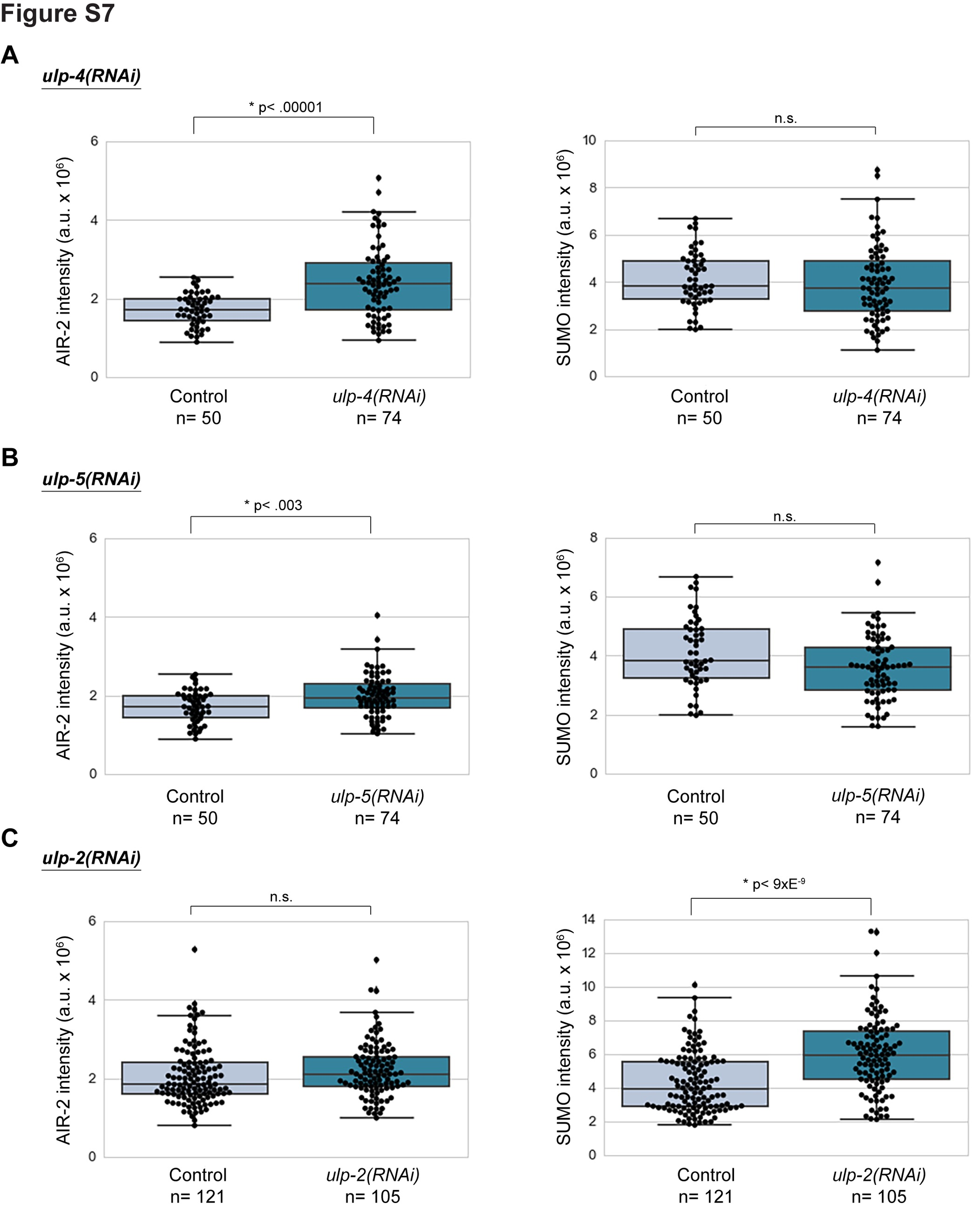

Supplement: S7 Fig — (A-C) Box plots showing GFP::AIR-2 intensity per RC (left) or mCherry::SUMO(GG) intensity per RC (right) during metaphase after vector control or long-term (5 day) ULP depletions; data points represent individual RCs. Box represents first quartile, median, and third quartile. Lines extend to data points within 1.5 interquartile range. Asterisks represent significant difference, n.s. = not significant (two-tailed t test). (A) ulp-4(RNAi) results in increased AIR-2 intensity while SUMO intensity is similar to the control. (B) ulp-5(RNAi) also results in increased AIR-2 intensity, while SUMO did not significantly change. (C) ulp-2(RNAi) results in increased SUMO intensity on RCs while AIR-2 intensity is similar to control RCs. (TIF) [file pgen.1007626.s007.tif]

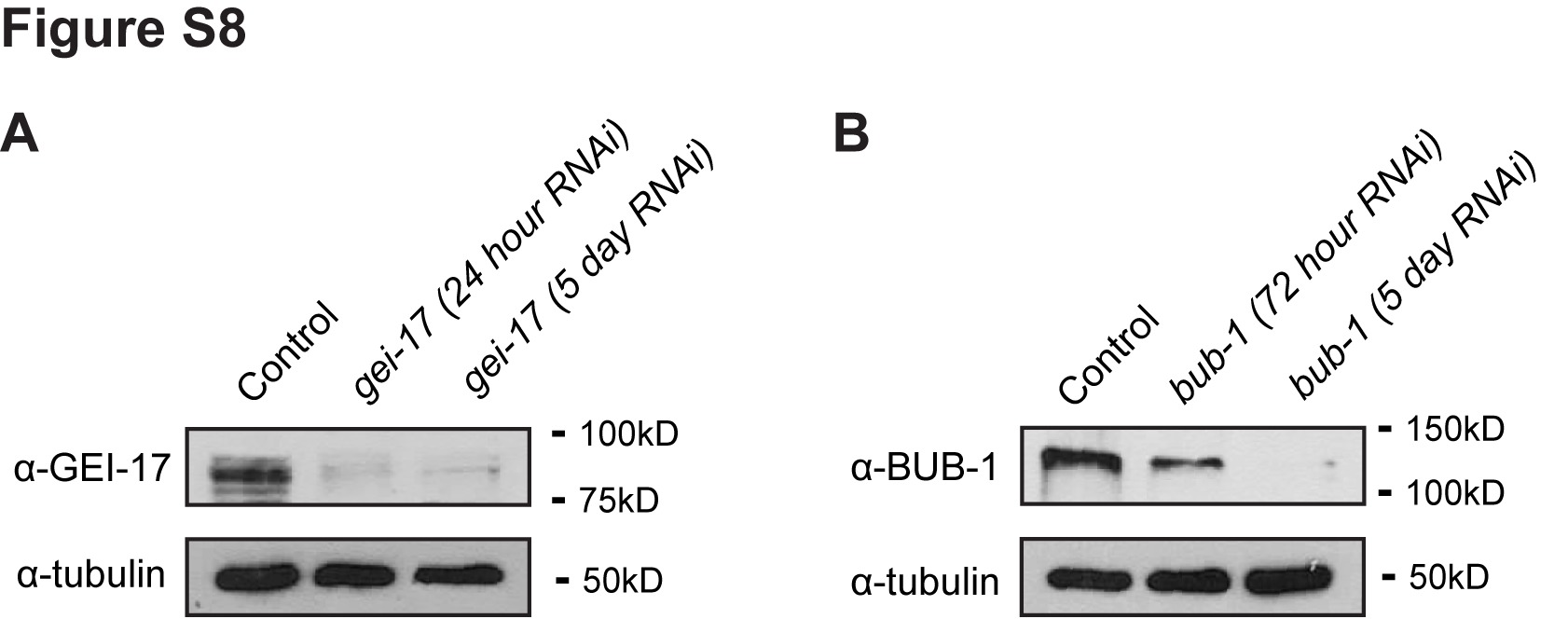

Supplement: S8 Fig — (A) Western blot showing the reduction in GEI-17 after vector control, 24 hour gei-17(RNAi), or 5 day gei-17(RNAi). (B) Western blot showing the reduction in BUB-1 after vector control, 72 hour bub-1(RNAi), or 5 day bub-1(RNAi). Tubulin is shown as the loading control. (TIF) [file pgen.1007626.s008.tif]
